# Supplementary material for: Older people’s experience of the partial lockdown during the COVID-19 pandemic in Switzerland: a cross-sectional study
Source: BMJ Open. 2023 Mar 23;13(3):e067167. doi: 10.1136/bmjopen-2022-067167 (PMC10039977; doi:10.1136/bmjopen-2022-067167)
Supplement: Supplementary data [file bmjopen-2022-067167supp001.pdf]

Supplementary material

Supplementary Table 1. Source of study variables in the questionnaires and time of measurement

1. Katz S, Ford AB, Moskowitz RW, Jackson BA, Jaffe MW. Studies of Illness in the Aged: The Index of ADL: A Standardized Measure of Biological and Psychosocial Function. JAMA. 1963 Sep 21;185(12):914–9.

2. Lawton MP, Brody EM. Assessment of Older People: Self-Maintaining and Instrumental Activities of Daily Living1. The Gerontologist. 1969 Oct 1;9(3_Part_1):179–86.

3. MacKay S, Ebert P, Harbidge C, Hogan DB. Fear of Falling in Older Adults: A Scoping Review of Recent Literature. Can Geriatr J. 2021 Dec 1;24(4):379–94.

4. Whooley MA. Screening for Depression—A Tale of Two Questions. JAMA Intern Med. 2016 Apr 1;176(4):436.

5. Boyer P, Bisserbe JC, Weiller E. How efficient is a screener? A comparison of the PRIME-MD patient questionnaire with the SDDS-PC screen. Int J Methods Psychiatr Res. 1998;7(1):27–32.

6. Sherbourne CD, Stewart AL. The MOS social support survey. Soc Sci Med 1982. 1991;32(6):705–14.

Abbreviation	Questionnaire	Time period of questionnaire completion
qlock	Lockdown questionnaire	17.04.2020 – 31.05.2020
q2016	Yearly questionnaire 2016	01.01.2016 – 31.12.2016
q2019	Yearly questionnaire 2019	01.01.2019 – 31.12.2019
q2020	Yearly questionnaire 2020	01.01.2020 – 31.12.2020
qbase	Baseline questionnaire	Time of enrollment for each cohort i.e.:<br>– Cohort 1: 01.01.2004 – 31.12.2004<br>– Cohort 2: 01.01.2009 – 31.12.2009<br>– Cohort 3: 01.01.2014 – 31.12.2014

**Outcome**	**Measure – Question in the lockdown questionnaire (qlock) April – May 2020**
**Experience of the lockdown**	qlock Q19 "To what extent is the lockdown currently difficult to cope with?"<br><br>With the choice of answers:<br><br>1. Not at all difficult<br>2. Slightly difficult<br>3. Very difficult or 4. Extremely difficult (grouped as one category)

**Characteristics**	**Measure – Questions in the yearly questionnaires (q2019, q2020, q2016), the baseline questionnaire (qbase) and lockdown questionnaire (qlock)**
**Age**	Mean (standard deviation)<br>2020 – birth year
**Gender**	Men / Women
**Education level**	qbase Q13 “What is the highest level of education you have completed?”<br><br>1. Compulsory schooling<br>2. Apprenticeship<br>3. Baccalaureate, high school diploma<br>4. Federal master's degree, professional diploma<br>5. University, college

	3 groups: 1 Basic compulsory/2 Apprenticeship/3 Post-compulsory schooling
**Frequency of going out of home (homebound status)**	q2019 Q31 (Q30 C2) "How frequently do you go out of your home?"<br>1. 5-7 days per week (non-homebound)<br>2. 3-4 days per week + 3. 1-2 days per week (grouped as one category: semi-homebound)<br>4. I almost never go out of my home (homebound)
**Living situation in 2020:**<br>**1. Living alone**<br>**2. Home type**<br>**3. Home location**<br>**4. Home characteristics**<br>**5. Having a pet animal**	1. qlock Q7 "What are your living arrangements during this lockdown period due to Covid?"<br>2 groups: answer 1 (alone) versus answer 2 (with … other persons)<br>2. qlock Q8a. "What are the characteristics of your living accommodation?"<br>apartment versus house<br>3. qlock Q8b. "What are the characteristics of your living accommodation?"<br>2 groups: in town; suburbs or country<br>4. qlock Q8c.+d. "What are the characteristics of your living accommodation?" balcony (yes/no) + terrace and/or garden (yes/no)<br>3 groups: no balcony/terrace/garden; having a balcony; having a terrace or a garden<br>5. q2020 Q61 (C2 56) "Do you live with a pet animal?" (yes/no)<br>q2020 Q62 (C2 57) "What animal(s) do you live with?"<br>a) cat(s)<br>b) dog(s)<br>c) other(s): specify<br>2 variables: Having a cat (yes/no) / Having a dog (yes/no)
**Indicators of low economic status (financial difficulties)**	1. q2019 Q51 (C2 Q45) "Do you currently receive state subsidies for your health insurance costs?" (yes/no)<br>2. q2019 Q52 (C2 Q46) "Do you currently receive complementary subsidies (PC) from the old age and survivor's insurance (AVS)?" (yes/no)<br>2 groups: absence of difficulties versus answering yes at one of the 2 questions
**Self-judgement of health status**	q2019, Q1 "In your opinion, what is your current state of health?"<br>answer 1+2 (very good and good) vs. 3+4+5 (average, poor, very poor)
**Comorbidities**	q2019 Q4: "During the past 12 months, were you treated, or did you suffer from any of the following health problems, diagnosed by a physician?"<br>-No illness (item 20) versus<br>-One of:<br>-Two or more of:<br>-Cardiac comorbidities: coronaropathy (3) + cardiac insufficiency (4) + valvular or cardiac muscle deficiency (5)<br>-TIA, stroke (6)<br>-Diabetes (7)<br>-Chronic pulmonary conditions (8) and asthma (9)

Märki-Germann D, *et al. BMJ Open* 2023; 13:e067167. doi: 10.1136/bmjopen-2022-067167

	-Osteoarticular conditions: osteoporosis (10) + fractures (11) + arthrosis or arthritis (12)<br>-Oncologic illness (13)<br>-Parkinson and Alzheimer (16) (17)
**Basic activities of daily living (BADL) and instrumental activities of daily living (IADL)**	q2019 Q43 (C2 Q40) "Do you have difficulties or receive help for the following activities due to physical, psychological, emotional or memory problems?" (bathing, getting dressed, toileting, transferring, feeding, continence (BADL); ability to use the telephone, to use transportations, prepare food, shop for groceries, do the housekeeping, prepare and take your medications, manage finances (IADL))<br><br>2 groups: independent BADL versus difficulties with at least 1 BADL (1)<br>2 groups: independent IADL versus difficulties with at least 1 IADL (2)
**Indicators of mobility:**<br>**1. Walking difficulties**<br>**2. Fear of falling**	1. q2019 Q35a (C2 Q34a) "Do you find it difficult to walk 100 meters for health reasons?" (yes/no)<br>2. q2019 Q37 (C2 Q36) "Do you fear to fall?" (yes/no) (3)
**Memory difficulties**	q2019 Q5 item 7, 8, 9 "Have you been disrupted since at least 6 months by memory gaps that affect your daily life or by concentration difficulties or decision-making difficulties?" (yes/no)
**Depressive symptoms (self-reported)**	1. q2019 Q17 "During the past 4 weeks, did you often feel sad, depressed or discouraged?" (yes/no)<br>2. q2019 Q18 "During the past 4 weeks, did you feel a loss of interest or pleasure in your usual activities?" (yes/no)<br><br>2 groups using Whooley's two questions (4): no symptoms versus presence of symptoms (sadness or apathy)
**Anxiety symptoms (self-reported)**	1. q2019 Q19 "During the past 4 weeks, did you often feel preoccupied and anxious?" (yes/no)<br>2. q2019 Q20 "During the past 4 weeks, how often did you feel calm, balanced, serene?" (rarely, never vs. other responses)<br><br>2 groups: no symptoms versus yes to question Q19 and/or sometimes/rarely/never to question Q20 adapted from the PRIME-MD screen (5)
**Feeling of loneliness**	q2019 Q21 "During the past 4 weeks, how often did you feel isolated?" (always, very often, often, sometimes, rarely, never)<br><br>2 groups: always/very often/often/sometimes versus rarely/never
**Usual social support**	1. q2016 Q44 (C2 Q32) With how many members of your family (partner, children, etc.) do you:<br>a) get in touch (visually or on the phone) at least once a month?<br>b) feel close enough to ask them for help when needed?<br>c) feel at ease to discuss private matters?<br><br>2. q2016 Q45 (C2 Q33) With how many friends or neighbors do you :<br>a) get in touch (visually or on the phone) at least once a month?<br>b) feel close enough to ask them for help when needed?<br>c) feel at ease to discuss private matters?<br><br>2 groups according to clinical cut point of Lubben Social Network Scale (2):<br>Socially supported (LSNS-6≥12) / Not socially supported (LSNS-6<12)

**Emotional Support**	1. q2016 Q46 (C2 Q34) How often can you count on someone to show you love or affection when needed? (always, very often, often, sometimes, rarely, never)<br>2. q2016 Q47 (C2 Q35) How often can you count on someone with whom you can share your intimate preoccupation when needed? (always, very often, often, sometimes, rarely, never)<br>3. q2016 Q48 (C2 Q36) How often can you rely on someone you love and for whom you are important? (always, very often, often, sometimes, rarely, never)<br><br>3 groups according to three items from the MOS social support survey (6): no emotional support declared as always, very often, often; 1-2 emotional support declared as always, very often, often; 3 emotional support declared as always, very often, often
**Familiarity with communication technology**	q2020 Q54 (C1 Q59) “Do you forgo certain activities because you are unfamiliar with technology?” (yes/no)
**Previous participation in group activities**	q2016 Q49 (C2 Q37) How many times a month do you participate in group activities (such as recreation activities, local activities, charitable associations, political or religious groups)<br><br>2 groups: 0 times versus > 0 times
**Death of a partner in the past year**	q2019 Q56 (C2 Q47) j: "During the past 12 months, did you have to face with the death of your spouse or partner?" (yes/no)

Supplementary Table 2. Comparisons of characteristics in participants included vs not included in the study

*P-values from Student's t-test for age and from Pearson's chi2 test for categorical variables.

	Total N=2755	Included N=2642	Not included N=113	P-value*
**Age, mean (sd)**	78.1 (4.2)	78.0 (4.2)	79.3 (4.6)	**.002**
**Gender, n (%)**				
Men	1093 (39.7)	1059 (40.1)	34 (30.1)	**.033**
Women	1662 (60.3)	1583 (59.9)	79 (69.9)	
**Education level, n (%)**				
Basic compulsory	438 (15.9)	404 (15.3)	34 (30.1)	**<.001**
Apprenticeship	1072 (39.0)	1032 (39.1)	40 (35.4)	
Post-compulsory schooling	1242 (45.1)	1203 (45.6)	39 (34.5)	
**Depressive symptoms, n (%)**				
No	1962 (72.0)	1916 (72.6)	46 (53.5)	**<.001**
Yes	763 (28.0)	723 (27.4)	40 (46.5)	
**Self-rated health, n (%)**				
Very good / good	1775 (65.1)	1734 (65.6)	41 (47.7)	**.001**
Average / poor / very poor	953 (34.9)	908 (34.4)	45 (52.3)	
**Living alone, n (%)**				
No	1527 (55.6)	1465 (55.6)	62 (55.4)	.956
Yes	1219 (44.4)	1169 (44.4)	50 (44.6)	
**Exp. of the lockdown, n (%)**				
Positive	596 (21.6)	569 (21.5)	27 (23.9)	
Neutral	1854 (67.3)	1791 (67.8)	63 (55.8)	
Negative	305 (11.1)	282 (10.7)	23 (20.3)	**.003**

Märki-Germann D, *et al. BMJ Open* 2023; 13:e067167. doi: 10.1136/bmjopen-2022-067167

Supplementary Table 3. Results of multivariable multinomial logistic regression investigating participants' characteristics associated with a positive ("not at all difficult") and negative ("very or extremely difficult") experience of the lockdown, using participants with a neutral ("slightly difficult") experience as reference group

	**Experience of the lockdown**					
	Positive (ref. neutral)			Negative (ref. neutral)		
	RRR	95% CI	P-value*	RRR	95% CI	P-value*
Socio-demographic						
**Gender**						
Men	ref.			ref.		
Women	**0.75**	**0.59 – 0.95**	**.016**	0.97	0.71 – 1.34	.873
**Financial difficulties**						
No	ref.			ref.		
Yes	1.08	0.84 – 1.40	.555	1.16	0.84 – 1.60	.353
Living environment						
**Living alone**						
No	ref.			ref.		
Yes	**1.93**	**1.52 – 2.46**	**<.001**	1.03	0.76 – 1.41	.836
**Home type**						
Apartment	ref.			ref.		
House	**1.49**	**1.03 – 2.16**	**.033**	0.67	0.34 – 1.32	.247
**Home characteristics**						
No balcony, terrace or garden	0.87	0.52 – 1.44	.578	0.63	0.32 – 1.25	.186
A balcony	ref.			ref.		
A terrace and/or a garden	1.05	0.79 – 1.40	.738	**0.66**	**0.44 – 0.99**	**.044**
Health status						
**Self-rated health**						
Very good / good	ref.			ref.		
Average / poor/ very poor	0.96	0.72 – 1.28	.761	1.23	0.88 – 1.74	.231
**Comorbidities**						
None	ref.			ref.		
1	0.79	0.62 – 1.01	.061	0.99	0.71 – 1.37	.952
2+	0.78	0.55 – 1.09	.144	0.71	0.46 – 1.08	.107
**BADL impairment**						
Independent	ref.			ref.		
Diff. / help for at least 1	1.13	0.80 – 1.58	.493	0.73	0.50 – 1.07	.104
**IADL impairment**						
Independent	ref.			ref.		
Diff. / help for at least 1	**0.78**	**0.61 – 0.99**	**.045**	1.40	1.00 – 1.98	.053
**Fear of falling**						
No	ref.			ref.		
Yes	**0.68**	**0.54 – 0.86**	**.001**	**1.52**	**1.07 – 2.15**	**.019**
**Memory difficulties**						
No	ref.			ref.		
Yes	1.04	0.74 – 1.47	.816	1.16	0.80 – 1.67	.440

Märki-Germann D, *et al. BMJ Open* 2023; 13:e067167. doi: 10.1136/bmjopen-2022-067167

**Depressive symptoms**						
No	ref.			ref.		
Yes	0.94	0.67 – 1.32	.737	1.44	0.99 – 2.10	.059
**Anxiety symptoms**						
No	ref.			ref.		
Yes	0.81	0.61 – 1.08	.151	1.13	0.79 – 1.61	.511
Social status						
**Feeling of loneliness**						
No	ref.			ref.		
Yes	**0.67**	**0.49 – 0.91**	**.011**	1.20	0.86 – 1.69	.281
**Socially supported**						
No	ref.			ref.		
Yes	**0.71**	**0.54 – 0.93**	**.012**	0.78	0.56 – 1.10	.158
**Emotional support**						
0	ref.			ref.		
1-2	1.05	0.65 – 1.71	.842	0.96	0.58 – 1.61	.889
3	1.36	0.89 – 2.08	.158	0.78	0.49 – 1.24	.293
**Previous participation in group activities**						
No	ref.			ref.		
Yes	**0.74**	**0.59 – 0.92**	**.007**	0.82	0.61 – 1.10	.193
**Having a dog**						
No	ref.			ref.		
Yes	1.05	0.67 – 1.64	.848	**0.32**	**0.11 – 0.90**	**.031**
**Unfamiliarity with technology**						
No	ref.			ref.		
Yes	**0.69**	**0.52 – 0.91**	**.008**	1.01	0.74 – 1.38	.952

Choice of variables: significant variables (threshold p<.05) in the bivariable multinomial logistic regression

Notes: N=2,246 complete cases (outcome distributed into 470/1,540/236)

RRR = relative risk ratio; CI = confidence interval. Reference: “slightly difficult”

* P-values from adjusted multinomial logistic regression.

Märki-Germann D, *et al. BMJ Open* 2023; 13:e067167. doi: 10.1136/bmjopen-2022-067167

Supplementary Table 3bis. Results from the multivariable multinomial logistic regression after multivariable imputation for missing data (sensitivity analysis).

	**Experience of the lockdown**					
	Not at all difficult (ref. slightly difficult)			Very or extremely difficult (ref. slightly difficult)		
	RRR	95% CI	P-value*	RRR	95% CI	P-value*
Socio-demographic						
**Gender**						
Men	ref.			ref.		
Women	**0.70**	**0.56 – 0.87**	**.001**	1.03	0.76 – 1.38	.858
**Financial difficulties**						
No	ref.			ref.		
Yes	1.10	0.87 – 1.38	.437	1.15	0.87 – 1.54	.326
Living environment						
**Living alone**						
No	ref.			ref.		
Yes	**1.90**	**1.52 – 2.37**	**<.001**	1.10	0.83 – 1.46	.519
**Home type**						
Apartment	ref.			ref.		
House	1.30	0.92 – 1.83	.131	0.70	0.37 – 1.32	.276
**Home characteristics**						
No balcony, terrace or garden	0.89	0.56 – 1.39	.596	0.62	0.33 – 1.13	.119
A balcony	ref.			ref.		
A terrace and/or a garden	1.10	0.85 – 1.42	.473	**0.62**	**0.43 – 0.90**	**.012**
Health status						
**Self-rated health**						
Very good / good	ref.			ref.		
Average / poor / very poor	0.99	0.77 – 1.29	.966	1.23	0.90 – 1.69	.198
**Comorbidities**						
None	ref.			ref.		
1	0.83	0.66 – 1.03	.094	1.03	0.76 – 1.40	.838
2+	0.89	0.65 – 1.21	.442	0.83	0.56 – 1.21	.325
**BADL impairment**						
Independent	ref.			ref.		
Diff. / help for at least 1	1.06	0.78 – 1.44	.713	0.77	0.55 – 1.10	.150
**IADL impairment**						
Independent	ref.			ref.		
Diff. / help for at least 1	**0.75**	**0.60 – 0.95**	**.016**	1.21	0.89 – 1.66	.227
**Fear of falling**						
No	ref.			ref.		
Yes	**0.73**	**0.59 – 0.90**	**.004**	**1.43**	**1.04 – 1.96**	**.029**
**Memory difficulties**						
No	ref.			ref.		
Yes	1.15	0.85 – 1.56	.366	1.09	0.77 – 1.53	.630
**Depressive symptoms**	ref.					
No	1.01	0.75 – 1.37		ref.	**1.16 – 2.31**	

Yes			.950	**1.63**		**.005**
**Anxiety symptoms**						
No	ref.			ref.		
Yes	0.78	0.60 – 1.01	.062	1.04	0.75 – 1.44	.829
Social status						
**Feeling of loneliness**						
No	ref.			ref.		
Yes	**0.62**	**0.47 – 0.83**	**.001**	1.07	0.78 – 1.46	.678
**Usual social support**						
No	ref.			ref.		
Yes	**0.73**	**0.57 – 0.94**	**.014**	0.80	0.58 – 1.12	.191
**Emotional support**						
0	ref.			ref.		
1-2	0.91	0.59 – 1.39	.651	0.94	0.59 – 1.50	.798
3	1.09	0.76 – 1.57	.646	0.77	0.51 – 1.18	.230
**Previous participation in group activities**						
No	ref.			ref.		
Yes	**0.79**	**0.65 – 0.97**	**.024**	0.83	0.64 – 1.09	.192
**Having a dog**						
No	ref.			ref.		
Yes	1.04	0.69 – 1.58	.849	**0.29**	**0.10 – 0.79**	**.016**
**Unfamiliarity with communication technology**						
No	ref.			ref.		
Yes	**0.66**	**0.52 – 0.85**	**.001**	1.12	0.85 – 1.49	.422

RRR relative risk ratio; CI confidence interval.

Reference: “slightly difficult” lockdown’s experience

* P-values from adjusted multinomial logistic regression.

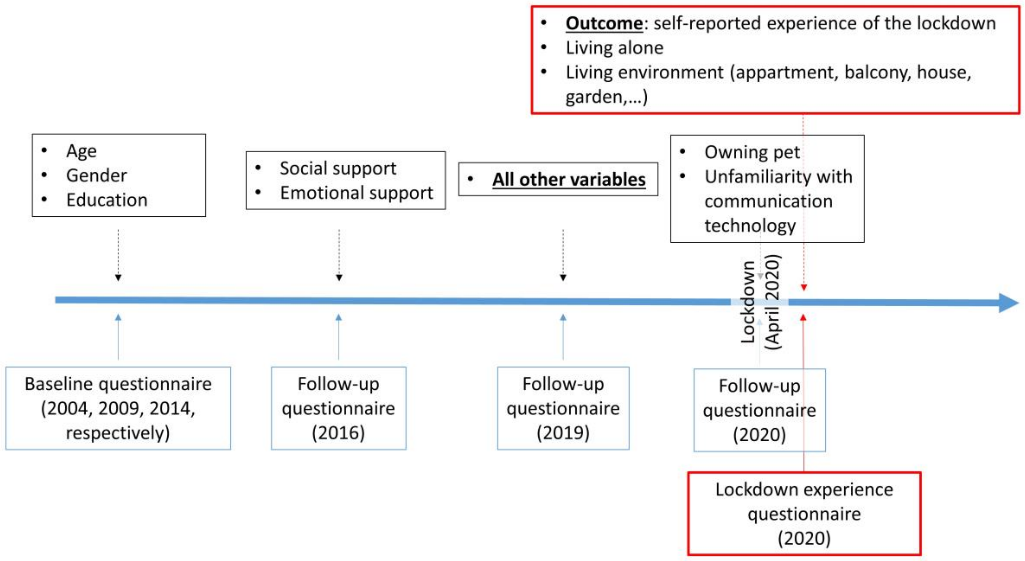

Supplementary Figure 1. Description of the sources of the study's variables and outcome
