# Supplementary material for: Older people’s experience of the partial lockdown during the COVID-19 pandemic in Switzerland: a cross-sectional study
Source: BMJ Open. 2023 Mar 23;13(3):e067167. doi: 10.1136/bmjopen-2022-067167 (PMC10039977; doi:10.1136/bmjopen-2022-067167)
Supplement: Author's manuscript [file bmjopen-2022-067167.draft_revisions.pdf]

BMJ Open is committed to open peer review. As part of this commitment we make the peer review history of every article we publish publicly available.

When an article is published we post the peer reviewers' comments and the authors' responses online. We also post the versions of the paper that were used during peer review. These are the versions that the peer review comments apply to.

The versions of the paper that follow are the versions that were submitted during the peer review process. They are not the versions of record or the final published versions. They should not be cited or distributed as the published version of this manuscript.

BMJ Open is an open access journal and the full, final, typeset and author-corrected version of record of the manuscript is available on our site with no access controls, subscription charges or pay-per-view fees (http://bmjopen.bmj.com).

If you have any questions on BMJ Open's open peer review process please email info.bmjopen@bmj.com

Older persons' experience of the partial lockdown during the COVID-19 pandemic

Journal:	*BMJ Open*
Manuscript ID	bmjopen-2022-067167
Article Type:	Original research
Date Submitted by the Author:	08-Aug-2022

Complete List of Authors:	Märki-Germann, Daphné; Lausanne University Hospital Department of Geriatrics and Geriatric Rehabilitation<br>Seematter-Bagnoud , Laurence; Center for Primary Care and Public Health, Epidemiology and Health systems; University Hospital of Lausanne, Geriatric Medicine and Rehabilitation<br>FUSTINONI, Sarah; Center for Primary Care and Public Health, Epidemiology and Health systems<br>SPALTENSTEIN, Julia; Lausanne University Hospital Department of Geriatrics and Geriatric Rehabilitation; Center for Primary Care and Public Health, Epidemiology and Health systems<br>Bula, Christophe; Lausanne University Hospital Department of Geriatrics and Geriatric Rehabilitation<br>HENCHOZ, Yves; Center for Primary Care and Public Health, Epidemiology and Health systems

Keywords:	COVID-19, GERIATRIC MEDICINE, MENTAL HEALTH

SCHOLARONE™
Manuscripts

I, the Submitting Author has the right to grant and does grant on behalf of all authors of the Work (as defined in the below author licence), an exclusive licence and/or a non-exclusive licence for contributions from authors who are: i) UK Crown employees; ii) where BMJ has agreed a CC-BY licence shall apply, and/or iii) in accordance with the terms applicable for US Federal Government officers or employees acting as part of their official duties; on a worldwide, perpetual, irrevocable, royalty-free basis to BMJ Publishing Group Ltd ("BMJ") its licensees and where the relevant Journal is co-owned by BMJ to the co-owners of the Journal, to publish the Work in this journal and any other BMJ products and to exploit all rights, as set out in our licence.

The Submitting Author accepts and understands that any supply made under these terms is made by BMJ to the Submitting Author unless you are acting as an employee on behalf of your employer or a postgraduate student of an affiliated institution which is paying any applicable article publishing charge ("APC") for Open Access articles. Where the Submitting Author wishes to make the Work available on an Open Access basis (and intends to pay the relevant APC), the terms of reuse of such Open Access shall be governed by a Creative Commons licence – details of these licences and which Creative Commons licence will apply to this Work are set out in our licence referred to above.

Other than as permitted in any relevant BMJ Author's Self Archiving Policies, I confirm this Work has not been accepted for publication elsewhere, is not being considered for publication elsewhere and does not duplicate material already published. I confirm all authors consent to publication of this Work and authorise the granting of this licence.

For peer review only

« Older persons' experience of the partial lockdown during the COVID-19 pandemic»

Daphné MÄRKI-GERMANN [1]

Laurence SEEMATTER-BAGNOUD, MD, MPH, PhD [1,2]

Sarah FUSTINONI, BSc [2]

Julia SPALTENSTEIN, MD [1,2]

Christophe BÜLA, MD [1]

Yves HENCHOZ, PhD[2]

(1) Service of Geriatric medicine and geriatric rehabilitation, Lausanne University Hospital, Lausanne, Switzerland

(2) Center for Primary Care and Public Health (Unisanté), University of Lausanne, Lausanne, Switzerland

Corresponding author:

Dr Daphné MÄRKI-GERMANN

Rue des Trois-Fontaines 11B, 1266 Duillier, SWITZERLAND

daphne.marki-germann@unil.ch

Word count : 3989

For peer review only - http://bmjopen.bmj.com/site/about/guidelines.xhtml

For peer review only

ABSTRACT

Objectives This study aimed to evaluate older persons' experience of a COVID-19 partial lockdown (March 16 - May 11, 2020) in Switzerland.

Setting and participants Community-dwelling participants of the Lausanne cohort Lc65+ in 2020, aged 71- 86 years (n=2642).

Design and outcome This study is nested within the Lc65+ longitudinal study. A specific COVID-19 questionnaire was sent on April 17, 2020, to evaluate participants' experience of the lockdown (outcome). Multinomial logistic regression models were used to determine the socio-demographic, living environment, health, and social factors associated with the outcome.

Results From 2,642 included participants (71–86 years old), 67.8% described the lockdown as "somewhat" difficult (reference group), 21.5% as "not at all" difficult (positive), 10.7% as "very or extremely" difficult (negative). The relative risk of a positive experience was higher for participants living alone (RRR=1.93, [95%CI] 1.52–2.46) or in a house (RRR=1.49, 1.03–2.16); lower in those who reported their fear of falling (RRR=0.68, 0.54–0.86), functional difficulties (RRR=0.78, 0.61–0.99), feeling of loneliness (RRR=0.67, 0.49–0.91), unfamiliarity with communication technologies (RRR=0.69, 0.52–0.91), usual social support (RRR=0.71, 0.50–0.93), previous participation to group activities (RRR=0.74, 0.59–0.92) and in women (RRR=0.75, 0.59–0.95). The relative risk of a negative experience was higher in participants with fear of falling (RRR=1.52, 1.07–2.15), and lower in those who had a terrace/garden (RRR=0.66, 0.44–0.99) and owned a dog (RRR=0.32, 0.11–0.90).

Conclusions Only one in ten participants experienced the lockdown as very or extremely difficult. Specific interventions could lessen the impact of a future similar situation.

For peer review only - http://bmjopen.bmj.com/site/about/guidelines.xhtml

Key words: COVID-19, partial lockdown, risk factors, sociodemographic factors, living environment

For peer review only

ARTICLE SUMMARY

Strengths and limitations of this study

- This study focused on the experience of the COVID-19 partial lockdown in a large sample of community-dwelling older persons.
- The design of the study allowed linking data collected during the lockdown with key characteristics routinely assessed in the Lc65+ longitudinal study, and to highlight the ones that were independently associated with a positive and a negative experience of the lockdown.
- Responses were obtained from postal – rather than online – questionnaires, allowing to include individuals who are uncomfortable with the use of communication technology.
- The most vulnerable participants (7.5% of total sample) had to be excluded from the study due to missing data or institutionalization.
- This study focused only on a specific type of lockdown, i.e., a partial lockdown.

For peer review only

INTRODUCTION

In Switzerland, the Federal Council ordered a partial lockdown period lasting from March 16, to May 11, 2020 to prevent the spread of the highly contagious SARS-CoV-2 virus. Strong recommendations to stay strictly at home were specifically directed toward older persons because of their increased vulnerability of severe COVID-19 infection and hospitalization.

Concerns rapidly emerged regarding the psychological impact of the lockdown. Early studies conducted during the first outbreak in China and the United States showed increased anxiety and depressive symptoms in the context of stay-at-home orders (1–3). Overall, nearly half of the general population reported a moderate to severe level of distress during the first outbreak of the epidemic (3–6). These early studies suggested that people aged 60 years and over were particularly at risk for such an adverse psychological impact (3).

The partial lockdown deprived socially active older persons who used to go outdoors of their usual contact with society. Older people who already were less active because of health problems also faced the fear of being infected through contacts with in-home care staff (7).

Several later studies conducted in other countries reported information about the early impact of the COVID-19 pandemic on anxiety and depressive symptoms in the general population (8–10). Results showed a significantly increasing prevalence of mental distress during the first months of the epidemic, in particular of anxio-depressive and post-traumatic stress disorder symptoms.

Other studies investigated the socio-demographic and health predictors of depression and anxiety symptoms associated with the epidemic and the stay-at-home orders in the general population (10,11). However, few specifically focused on the older population and none on the specific conditions of a partial lockdown. For instance, it remains unclear whether age itself is

For peer review only - http://bmjopen.bmj.com/site/about/guidelines.xhtml

a risk factor for a negative experience or, reversely, whether it could be a protective factor due to resilience and accommodative strategies (12,13). Similarly, the potential mediating effect of mobility and functional capacities has not been investigated.

A study conducted in Zürich, Switzerland, in 99 persons aged 65 years and over showed that their level of well-being decreased as their feeling of loneliness increased during the first 4 weeks of the partial lockdown (14). Another Swiss study conducted in the Ticino Canton in 19 community-dwelling older persons revealed the latter's ambivalent feelings about the lockdown; some of them expressed their feeling of exclusion because older persons were categorized as vulnerable (15).

This study primarily aimed to evaluate how a large sample of older persons experienced the partial lockdown during the first wave of the SARS-CoV-2 epidemics in Switzerland. Our main hypothesis was that most participants would report a neutral feeling, judging this period as somewhat difficult, whereas two smaller subgroups would report more extreme feelings and consider their experience as either negative (judging the period as very difficult) or positive (judging the period as not difficult at all).

A second aim was to study participants' characteristics associated with these more extreme feelings about their experience of the partial lockdown. More specifically, this study sought to investigate the association of four categories of participants' characteristics (socio-demographic, living environment, health status and social status) with a positive, respectively negative, experience of the lockdown.

Our hypotheses were that vulnerability factors (older age, lower education, higher comorbidities, as well as functional, cognitive, and affective impairments) would be associated with a higher risk of a negative experience. By contrast, we hypothesized that participants living in a more comfortable environment (in a house, with access to a garden or a balcony) would be

For peer review only - http://bmjopen.bmj.com/site/about/guidelines.xhtml

more likely to report a positive experience. Finally, our hypotheses were mixed regarding social factors, as we expected that participants having less usual social support would be more likely to report a negative experience, whereas those owning pet animals might report a positive experience.

For peer review only

For peer review only - http://bmjopen.bmj.com/site/about/guidelines.xhtml

METHODS

Study design and participants

This study is nested within the Lausanne cohort 65+ (Lc65+) longitudinal study (16,17), initially designed to investigate the development of age-related frailty. Three samples of non-institutionalized individuals living in Lausanne (Switzerland) were successively enrolled at the same age of 65 to 70 years in 2004 (birth years 1934–1938), 2009 (1939–1943), and 2014 (1944–1948).

Data on participants' characteristics stem from the recruitment (2004, 2009, and 2014, respectively) and follow-up (2016, 2019, and 2020) questionnaires. A separate COVID-19 specific questionnaire was sent to the participants on April 17, 2020. The questionnaires relating to their experience of the partial lockdown were completed during the lockdown to exclude memory bias. As a simplification measure, the "partial lockdown" will be referred to as "lockdown" below.

The Lc65+ study protocol, including the COVID-19 specific questionnaire, was approved by the ethical committee for human's research in the canton of Vaud (Protocol No. 19/04). Written informed consent was obtained from the participants.

Measurement of self-reported experience of the lockdown (outcome)

The study's main outcome derived from participants' answer to the following question in the COVID-19 specific questionnaire: "To what extent is the lockdown currently difficult to live with?"

According to their answers, the participants' experience of the lockdown was defined as: a) **Positive** in participants who answered "not difficult at all"; b) **Neutral** (defined as the reference

For peer review only - http://bmjopen.bmj.com/site/about/guidelines.xhtml

group) in participants who answered “somewhat difficult”; and c) **Negative** in those who answered “very difficult” or “extremely difficult”. Thus, the term “positive” refers to participants who reported a more positive experience of the lockdown than other participants and does not imply that the lockdown was a positive experience in itself.

Measurement of participants’ characteristics

Variables with hypothesized associations with participants’ experience of the lockdown (outcome) were identified a priori in the four following domains:

Socio-demographic variables included: age (at the end of year 2020), gender, education level (low: compulsory school; medium: apprenticeship; high: college, university degree or equivalent), and financial difficulties (entitlement to supplementary benefits to old-age insurance).

Living environment variables included: living alone versus with other persons, home type (apartment versus house), home characteristics (having a balcony, a terrace/garden, or none of the above), home location (countryside/suburbs versus urban area).

Health status variables included: self-rated health, the presence or absence of comorbidities, impairment in basic and instrumental activities of daily living (BADL, IADL), mobility indicators (walking difficulties, fear of falling), the fact of reporting memory difficulties or depressive/anxiety symptoms, and homebound status.

Social status variables included: usual social support (using elements of the revised Lubben social network scale (18)), emotional support, the feeling of loneliness, the recent death of the partner, the familiarity with communication technology, group activity participation, and owning a dog and/or a cat.

For peer review only - http://bmjopen.bmj.com/site/about/guidelines.xhtml

Most information was retrieved from the 2019 questionnaire. When they were unavailable that year, data from the most recent follow-up was used instead (for details see Supplementary – Table 1, Supplementary – Figure 1).

Statistical analysis

Participants' characteristics were first compared in bivariable analyses between participants with positive, neutral, and negative self-reported experience of the lockdown, respectively, using parametric and non-parametric tests (i.e., Chi-square and Fisher exact tests) according to the variables' distribution. Multinomial logistic regression analyses were then performed to investigate the bivariable and multivariable associations between participants' characteristics and a positive or negative experience of the lockdown, using participants with neutral experience as the reference group. Variables that proved statistically significant ($p<.05$) in bivariable analysis were included as candidate variables in the multivariable model.

Tests were then carried out on interactions between gender and several specific characteristics (fear of falling, living alone, depressive and anxiety symptoms), as well as between living alone and usual social support, instrumental activities of daily living, self-rated health, emotional support, or unfamiliarity with communication technologies.

To assess the effect of missing data, a sensitivity analysis was performed using a multivariate imputation approach. Assuming that covariables were missing at random, missing values were imputed iteratively by using chained equations, generating fifty complete datasets. A multivariable analysis was run on each multiply-imputed dataset, leading to fifty sets of coefficients and standard errors. The estimates were then pooled according to Rubin's combination rules (19).

For peer review only - http://bmjopen.bmj.com/site/about/guidelines.xhtml

Patient and public involvement Patients and/or the public were not involved in the design, or conduct, or reporting, or dissemination plans of this research.

For peer review only

For peer review only - http://bmjopen.bmj.com/site/about/guidelines.xhtml

RESULTS

Overall, 2,756 of the 3,087 (response rate 89.3%) contacted participants returned their completed COVID-19 questionnaires and 2,755 replied to the specific question about their experience of the lockdown. Figure 1 details the process of the population selection. After the exclusion of institutionalized participants (n=5), and of those who did not complete the previous questionnaires (n=108), analyses were conducted on a total of 2,642 participants (85.6% of sent questionnaires).

Compared to included participants (Supplementary – Table 2), those excluded (n=113) were older (P=.002), more often women (P=.033), had a lower education level (P<.001), more frequently reported depressive symptoms (P<.001), poor self-rated health (P=.001), and a "very difficult" or "extremely difficult" experience of the lockdown (P=.003).

Table 1 details participants' characteristics and provides the results of the comparisons across study groups according to their experience of the lockdown. Overall, 1,791 (67.8%) participants reported a "somewhat difficult" (neutral, reference group) experience of the lockdown, 569 (21.5%) participants reported this experience as "not at all difficult" (positive) and 282 (10.7%) as "very or extremely difficult" (negative).

Table 1. Characteristics of the study population and comparisons between participants with positive, neutral, and negative experience of the lockdown

	Experience of the lockdown*				
	Total population N = 2642	Positive n = 569 (21.5)	Neutral (reference) n = 1791 (67.8)	Negative n = 282 (10.7)	P-value†
Socio-demographic					
**Age, mean (sd)**	78.0 (4.2)	77.8 (4.2)	78.1 (4.1)	78.1 (4.4)	.434

For peer review only - http://bmjopen.bmj.com/site/about/guidelines.xhtml

**Gender, n (%)**					
Men	1059 (40.1)	272 (47.8)	694 (38.7)	93 (33.0)	**<.001**
Women	1583 (59.9)	297 (52.2)	1097 (61.3)	189 (67.0)	
**Education level, n (%)**					
Basic compulsory	404 (15.3)	89 (15.7)	261 (14.6)	54 (19.2)	.245
Apprenticeship	1032 (39.1)	210 (37.0)	712 (39.8)	110 (39.0)	
Post-compulsory schooling	1203 (45.6)	268 (47.3)	817 (45.6)	118 (41.8)	
**Financial difficulties, n (%)**					
No	1960 (74.2)	420 (73.8)	1348 (75.3)	192 (68.1)	**.035**
Yes	681 (25.8)	149 (26.2)	442 (24.7)	90 (31.9)	
Living environment					
**Living alone, n (%)**					
No	1465 (55.6)	288 (50.8)	1037 (58.1)	140 (49.7)	**.001**
Yes	1169 (44.4)	279 (49.2)	748 (41.9)	142 (50.3)	
**Home type, n (%)**					
Apartment	2298 (87.2)	475 (83.9)	1556 (87.0)	267 (94.7)	**<.001**
House	338 (12.8)	91 (16.1)	232 (13.0)	15 (5.3)	
**Home location, n (%)**					
City	2061 (78.7)	428 (76.2)	1401 (78.7)	232 (83.4)	.052
Suburbs/rural areas	559 (21.3)	134 (23.8)	379 (21.3)	46 (16.6)	
**Home characteristics, n (%)**					
No balcony / terrace / garden	140 (5.3)	29 (5.1)	98 (5.5)	13 (4.6)	**<.001**
A balcony	1684 (63.9)	339 (59.9)	1128 (63.0)	217 (77.0)	
A terrace and/or a garden	813 (30.8)	198 (35.0)	563 (31.5)	52 (18.4)	
Health status					
**Self-rated health, n (%)**					
Very good / good	1734 (65.6)	419 (73.6)	1173 (65.5)	142 (50.3)	**<.001**
Average / poor / very poor	908 (34.4)	150 (26.4)	618 (34.5)	140 (49.7)	
**Comorbidities, n (%)**					
None	1150 (43.7)	288 (51.1)	760 (42.6)	102 (36.4)	**.001**
1	981 (37.3)	189 (33.5)	675 (37.8)	117 (41.8)	
2+	498 (18.9)	87 (15.4)	350 (19.6)	61 (21.8)	
**BADL impairment‡, n (%)**					
Independent	2172 (82.4)	486 (85.9)	1467 (82.0)	219 (77.9)	**.013**
Difficulty or help for at least 1	464 (17.6)	80 (14.1)	322 (18.0)	62 (22.1)	
**IADL impairment ‖, n (%)**					
Independent	1239 (47.5)	329 (59.4)	819 (46.2)	91 (32.6)	**<.001**
Difficulty or help for at least 1	1367 (52.5)	225 (40.6)	954 (53.8)	188 (67.4)	
**Walking difficulty, n (%)**					
No	2285 (87.1)	493 (87.4)	1554 (87.3)	238 (85.0)	.546
Yes	339 (12.9)	71 (12.6)	226 (12.7)	42 (15.0)	
**Fear of falling, n (%)**					
No	1136 (43.1)	316 (55.4)	744 (41.6)	76 (27.1)	**<.001**
Yes	1502 (56.9)	253 (44.6)	1044 (58.4)	205 (72.9)	
**Homebound status, n (%)**					
Non-homebound	2046 (77.4)	437 (76.8)	1387 (77.4)	222 (78.7)	.537
Semi-homebound	577 (21.8)	125 (22.0)	394 (22.0)	58 (20.6)	
Homebound	19 (0.7)	7 (1.2)	10 (0.6)	2 (0.7)	

For peer review only - http://bmjopen.bmj.com/site/about/guidelines.xhtml

**Memory difficulties, n (%)**						
	No	2218 (84.3)	492 (86.9)	1507 (84.6)	229 (77.7)	**.002**
	Yes	412 (15.7)	74 (13.1)	275 (15.4)	63 (22.3)	
**Depressive symptoms, n (%)**						
	No	1916 (72.6)	460 (81.0)	1302 (72.8)	154 (54.6)	**<.001**
	Yes	723 (27.4)	108 (19.0)	487 (27.2)	128 (45.4)	
**Anxiety symptoms, n (%)**						
	No	1693 (64.4)	423 (74.7)	1130 (63.4)	140 (49.7)	**<.001**
	Yes	938 (35.6)	143 (25.3)	653 (36.6)	142 (50.3)	
Social status						
**Feelings of loneliness, n (%)**						
	No	1954 (74.1)	472 (83.2)	1311 (73.2)	171 (60.9)	**<.001**
	Yes	684 (25.9)	95 (16.8)	479 (26.8)	110 (39.1)	
**Usual social support, n (%)**						
	No	596 (24.8)	138 (26.7)	374 (22.8)	84 (33.5)	**.001**
	Yes	1811 (75.2)	378 (73.3)	1266 (77.2)	167 (66.5)	
**Emotional support, n (%)**						
	0	255 (9.7)	51 (9.0)	162 (9.1)	42 (15.0)	**.001**
	1 - 2	398 (15.2)	74 (13.1)	269 (15.1)	55 (19.6)	
	3	1974 (75.1)	440 (77.9)	1351 (75.8)	183 (65.4)	
**Previous participation in group activities, n (%)**						
	No	1112 (42.7)	263 (47.0)	716 (40.5)	133 (48.0)	**.004**
	Yes	1495 (57.3)	297 (53.0)	1054 (59.5)	144 (52.0)	
**Having a dog, n (%)**						
	No	2508 (95.1)	533 (93.7)	1697 (95.0)	278 (98.6)	**.007**
	Yes	130 (4.9)	36 (6.3)	90 (5.0)	4 (1.4)	
**Having a cat, n (%)**						
	No	2301 (87.2)	488 (85.8)	1577 (88.2)	236 (83.7)	.051
	Yes	337 (12.8)	81 (14.2)	210 (11.8)	46 (16.3)	
**Unfamiliarity with communication technology, n (%)**						
	No	1883 (72.1)	458 (81.4)	1252 (70.8)	173 (61.8)	**<.001**
	Yes	729 (27.9)	105 (18.6)	517 (29.2)	107 (38.2)	
**Death of partner in the previous year, n (%)**						
	No	2561 (97.8)	550 (97.9)	1735 (97.6)	276 (98.6)	.607
	Yes	58 (2.2)	12 (2.1)	42 (2.4)	4 (1.4)	

* Self-reported experience defined as “Positive”, “Neutral”, and “Negative” in participants reporting having experienced the lockdown as « not at all difficult », « somewhat difficult », and « very or extremely difficult », respectively.

†P-values from ANOVA test for age, and from Pearson’s chi2-test or Fischer exact test when expected frequencies <5, for categorical variables.

‡BADL: basic activities of daily living (bathing, dressing, toileting, transferring, feeding and continence)

‖ IADL: instrumental activities of daily living (ability to use the telephone, to use transportations, prepare food, groceries, do the housekeeping, handle medications and manage finances).

For peer review only - http://bmjopen.bmj.com/site/about/guidelines.xhtml

Missing values (n): Education level (3), Financial difficulties (1), Living alone (8), Home type (6), Home location (22), Home characteristics (5), Comorbidities (13), BADL impairment (6), IADL impairment (36), Walking difficulty (18), Fear of falling (4), Memory difficulties (12), Depressive symptoms (3), Anxiety symptoms (11), Feelings of loneliness (4), Usual social support (235), Emotional support (15), Previous participation in group activities (35), Having a dog (4), Having a cat (4), Unfamiliarity with communication technology (30), Death of partner in the previous year (23).

Participants' experience of the lockdown did not differ according to their age (P=.434) and education level (P=.245). However, several other characteristics differed between study groups, some of which showed monotonic increasing or decreasing trends across groups, whereas other showed U- or inverted U-shaped associations.

For instance, among **socio-demographic** characteristics**,** the proportion of women steadily increased across participants who reported a positive, neutral and negative experience of the lockdown (P<.001). By contrast, the proportion of participants experiencing financial difficulties was lower in the group reporting a neutral experience than in both other groups (P=.035).

Most **living environment** characteristics also showed significant associations with steadily decreasing proportions of participants living in a house (P <.001) and having a terrace and/or garden (P<.001) across groups reporting positive, neutral and negative experience. By contrast, the proportion of participants living alone was lower in participants with neutral experience than in both other groups (P=.001).

Similarly, most **health status** characteristics showed very important and significant inter-groups differences. The proportions of participants with poor self-rated health (P<.001), comorbidities (P=.001), BADL impairment (P=.013), IADL impairment (P<.001), fear of falling (P<.001), memory difficulties (P=.002), depressive symptoms (P<.001) and anxiety

For peer review only - http://bmjopen.bmj.com/site/about/guidelines.xhtml

symptoms (P<.001) increased across the groups that reported a positive, neutral and negative experience of the lockdown.

Finally, **social status** characteristics also showed monotonic trends with increasing proportions of participants feeling lonely (P<.001), without usual social (P=.001) and emotional (P=.001) support, not having a dog (P=.007) and unfamiliar with communication technology (P<.001) across the positive, neutral, and negative groups. The lack of previous participation in group activities (P=.004) was the only characteristic with a lower proportion among the neutral group.

Table 2 shows the results of the **bivariate multinomial logistic regression**, comparing the groups reporting a positive experience and a negative experience to the neutral group (reference group).

Table 2. Results from bivariable multinomial logistic regression investigating participants' characteristics associated with a positive ("not at all difficult") and a negative ("very or extremely difficult") experience of the lockdown, using participants with a neutral ("slightly difficult") experience as reference group

	Experience of the lockdown					
	Positive (ref. neutral)			Negative (ref. neutral)		
	RRR	95% CI	P-value*	RRR	95% CI	P-value*
Socio-demographic						
**Age**	0.99	0.96 – 1.01	.223	1.00	0.97 – 1.03	.840
**Gender**						
Men	ref.			ref.		
Women	**0.69**	**0.57 – 0.84**	**<.001**	1.29	0.99 – 1.68	.064
**Education level**						
Basic compulsory	ref.			ref.		
Apprenticeship	0.86	0.65 – 1.15	.319	0.75	0.52 – 1.07	.107
Post-compulsory schooling	0.96	0.73 – 1.27	.784	**0.70**	**0.49 – 0.99**	**.045**
**Financial difficulties**						
No	ref.			ref.		
Yes	1.08	0.87 – 1.34	.474	**1.43**	**1.09 – 1.88**	**.010**
Living environment						
**Living alone**						
No	ref.			ref.		
Yes	**1.34**	**1.11 – 1.62**	**.002**	**1.41**	**1.09 – 1.81**	**.008**

For peer review only - http://bmjopen.bmj.com/site/about/guidelines.xhtml

**Home type**						
Apartment	ref.			ref.		
House	1.28	0.99 – 1.67	.062	**0.38**	**0.22 – 0.65**	**<.001**
**Home location**						
City	ref.			ref.		
Suburbs/rural areas	1.16	0.92– 1.45	.203	0.73	0.52 – 1.03	.070
**Home characteristics**						
No balcony, terrace or garden	0.98	0.64 – 1.52	.944	0.69	0.38 – 1.25	.222
A balcony	ref.			ref.		
A terrace and/or a garden	1.17	0.96 – 1.43	.128	**0.48**	**0.35 – 0.66**	**<.001**
Health status						
**Self-rated health**						
Very good / good	ref.			ref.		
Average / poor / very poor	**0.68**	**0.55 – 0.84**	**<.001**	**1.87**	**1.45 – 2.41**	**<.001**
**Comorbidities**						
None	ref.			ref.		
1	**0.74**	**0.60 – 0.91**	**.005**	1.29	0.97 – 1.72	.079
2+	**0.66**	**0.50 – 0.86**	**.002**	1.30	0.92 – 1.83	.134
**BADL impairment**						
Independent	ref.			ref.		
Diff. / help for at least 1	**0.75**	**0.58 – 0.98**	**.034**	1.29	0.95 – 1.75	.104
**IADL impairment**						
Independent	ref.			ref.		
Diff. / help for at least 1	**0.59**	**0.48 – 0.71**	**<.001**	**1.77**	**1.36 – 2.32**	**<.001**
**Walking difficulty**						
No	ref.			ref.		
Yes	0.99	0.74 – 1.32	.946	1.21	0.85 – 1.73	.287
**Fear of falling**						
No	ref.			ref.		
Yes	**0.57**	**0.47 – 0.69**	**<.001**	**1.92**	**1.45 – 2.54**	**<.001**
**Homebound status**						
Non-homebound	ref.			ref.		
Semi-homebound	1.01	0.80 – 1.26	.953	0.92	0.67 – 1.25	.597
Homebound	2.22	0.84 – 5.87	.107	1.25	0.27 – 5.74	.775
**Memory difficulties**						
No	ref.			ref.		
Yes	0.82	0.63 – 1.09	.170	**1.58**	**1.16 – 2.15**	**.004**
**Depressive symptoms**						
No	ref.			ref.		
Yes	**0.63**	**0.50 – 0.79**	**<.001**	**2.22**	**1.72 – 2.87**	**<.001**
**Anxiety symptoms**						
No	ref.			ref.		
Yes	**0.59**	**0.47 – 0.72**	**<.001**	**1.76**	**1.36 – 2.26**	**<.001**
Social status						
**Feelings of loneliness**						
No	ref.			ref.		
Yes	**0.55**	**0.43 – 0.70**	**<.001**	**1.76**	**1.36 – 2.29**	**<.001**
**Usual social support**	ref.					
No	0.81	0.65 – 1.01		ref.	**0.44 – 0.78**	

For peer review only

For peer review only - http://bmjopen.bmj.com/site/about/guidelines.xhtml

	Yes			.067	**0.59**		**<.001**
**Emotional support**							
	0	ref.			ref.		
	1-2	0.87	0.58 – 1.31	.515	0.79	0.50 – 1.23	.297
	3	1.03	0.74 – 1.44	.841	**0.52**	**0.36 – 0.76**	**<.001**
**Previous participation in group activities**							
	No	ref.			ref.		
	Yes	**0.77**	**0.63 – 0.93**	**.007**	**0.74**	**0.57 – 0.95**	**.018**
**Having a dog**							
	No	ref.			ref.		
	Yes	1.27	0.85 – 1.90	.234	**0.27**	**0.10 - 0.74**	**.011**
**Having a cat**							
	No	ref.			ref.		
	Yes	1.25	0.95 – 1.64	.117	**1.46**	**1.03 – 2.07**	**.031**
**Unfamiliarity with communication technology**							
	No	ref.			ref.		
	Yes	**0.56**	**0.44 – 0.70**	**<.001**	**1.50**	**1.15 – 1.95**	**.003**
**Death of partner in the previous 12 months**							
	No	ref.			ref.		
	Yes	0.90	0.47 – 1.72	.754	0.60	0.21 – 1.68	.331

Notes: RRR relative risk ratio; CI confidence interval.
* P-values from multinomial logistic regression.

For peer review only

The relative risk of reporting a **positive experience of the lockdown** – rather than a neutral one – was 34% **higher** among participants living alone (RRR 1.34, 95%CI 1.11–1.62), but 31% **lower** among women (RRR 0.69, 95%CI 0.57–0.84). It was also reduced in participants with average, poor or very poor self-rated health, comorbidities, impairment in BADL and IADL, fear of falling, depressive symptoms, anxiety symptoms, a feeling of loneliness, previous participation to group activities and unfamiliarity with communication technology (P<.05 for all associations).

The relative risk of reporting a **negative experience of the lockdown** – rather than a neutral one – **increased** among participants who reported financial difficulties (RRR 1.43, 95%CI 1.09–1.88), but also in participants living alone, rating their health as average, poor or very poor, reporting IADL impairment, fear of falling, memory difficulties, depressive symptoms,

For peer review only - http://bmjopen.bmj.com/site/about/guidelines.xhtml

anxiety symptoms, a feeling of loneliness, having a cat, and unfamiliarity with communication technology (P<.05 for all associations). On the contrary, the relative risk **decreased** among participants with a post-compulsory education level (RRR 0.70, 95%CI 0.49–0.99), but also in participants owning a house, a terrace and/or a garden, benefiting from usual social support, reporting at least 3 sources of emotional support, previous participation to group activities and owning a dog (P<.05 for all associations).

Of note, some characteristics showed significant associations in a consistent direction, with increased relative risks of a negative experience on the one hand, and decreased relative risks of a positive experience on the other hand. For instance, participants who reported fear of falling had a 43% lower relative risk of reporting a positive experience (RRR 0.57, 95%CI 0.47–0.69) and were almost twice as likely to report a negative experience (RRR 1.92, 95%CI 1.45–2.54) of the lockdown compared to those without any fear of falling. Similar associations with both significant positive and negative experience were also observed for self-rated health, IADL impairment, depressive symptoms, anxiety symptoms, feelings of loneliness, and unfamiliarity with communication technology.

Two characteristics showed a U-shaped association with the experience of the lockdown. Participants living alone had a higher relative risk to report both a positive (RRR 1.34, 95%CI 1.11–1.62) and a negative (RRR 1.41, 95%CI 1.09–1.81) experience compared to a neutral one. Inversely, previous participation in group activities (reported by 59.5% of the total population) was associated with decreased relative risks of reporting a positive (RRR 0.77, 95%CI 0.63–0.93) or a negative (RRR 0.74, 95%CI 0.57–0.95) experience.

Figure 2 shows the results of the **multivariable multinomial logistic regression**, (for more details, see Supplementary – Table 3). The relative risk of reporting a **positive experience** – rather than a neutral one – remained **higher** among participants living alone (RRR 1.93, 95%CI

For peer review only - http://bmjopen.bmj.com/site/about/guidelines.xhtml

1.52–2.46) and those who owned a house (RRR 1.49, 95%CI 1.03–2.16), and **lower** among women (RRR 0.75, 95%CI 0.59–0.95), participants with IADL impairment (RRR 0.78, 95%CI 0.61–0.99), fear of falling (RRR 0.68, 95%CI 0.54–0.86), feelings of loneliness (RRR 0.67, 95%CI 0.49–0.91), those who received usual social support (RRR 0.71, 95%CI 0.54–0.93), and those with previous participation in group activities (RRR 0.74, 95%CI 0.59–0.92), and unfamiliar with communication technology (RRR 0.69, 95%CI 0.52–0.91).

Few characteristics remained independently associated with a **negative experience** of the lockdown. The relative risk for the latter – compared to a neutral one – **increased** among participants with fear of falling (RRR 1.52, 95%CI 1.07–2.15), and **decreased** among those who had a terrace and/or a garden (RRR 0.66, 95%CI 0.44–0.99), or owned a dog (RRR 0.32, 95%CI 0.11–0.9).

In the multivariable model, the fear of falling was the only factor that remained significantly associated in **a consistent direction**, with an increased relative risk of a negative experience (RRR 1.52, 95%CI 1.07–2.15) and a decreased relative risk of a positive experience (RRR 0.68, 95%CI 0.54–0.86) compared to the neutral group.

None of the tested interactions were significant. The sensitivity analysis conducted using the multivariate imputation approach produced very similar results (see Supplementary – Table 3bis).

For peer review only - http://bmjopen.bmj.com/site/about/guidelines.xhtml

DISCUSSION

This study examined the experience of the COVID-19 partial lockdown in a community-dwelling Swiss older population. Its unique contribution was to highlight which participants' characteristics were independently associated with a positive and a negative experience, respectively. Results confirmed our initial hypothesis that the majority of participants (about two-thirds) would report a neutral experience and assess this period as "somewhat difficult". By contrast, only about one in ten older persons reported a negative experience, assessing the lockdown as "very" or "extremely" difficult. At the other end of the spectrum, more than a fifth reported a rather positive experience, assessing the lockdown as "not difficult at all". Most of the observed associations were in line with our hypotheses: participants who lived in a more spacious environment (living in a house) and/or with outdoor access (having a terrace or a garden) were significantly less likely than their counterparts to report a negative rather than a neutral experience. Reversely, participants with health problems (fear of falling and functional difficulties) as well as those with social vulnerability (reporting a feeling of loneliness, usually receiving social support, and unfamiliar with communication technology) were significantly less likely to report a positive experience.

Several notable associations were rather unexpected. For instance, contrary to our initial hypothesis, living alone turned out to be associated with a higher relative risk of a positive experience. This result contrasts with another study that identified living alone as a vulnerability factor during a lockdown (20). The possibility to adjust for the feeling of loneliness might explain the current study's finding in that regard. Indeed, participants who expressed a feeling of loneliness were significantly less likely than their counterparts to report a positive rather than a neutral experience –a finding that is consistent with results of another Swiss study (14). This

For peer review only - http://bmjopen.bmj.com/site/about/guidelines.xhtml

hypothesis is further supported by the observation that participants with usual social support and previous participation in group activities were more likely to report a negative experience of the lockdown. Overall, these results suggest that older participants who were socially more active and involved, and those who already felt lonely were less able to cope with the lockdown than those already living alone before the pandemic. Indeed, older persons who still live alone in their home tend to be more mobile, more independent and autonomous, and may not be particularly prone to social isolation (21). However, this hypothesis is not supported in this study. Indeed 36.3% of participants living alone reported a feeling of loneliness, compared to 17.6% of those living with others. In this regard, the observed relationship between participants' unfamiliarity with communication technology and a reduced chance of reporting a positive experience of the lockdown is of particular interest. Maintaining the link with their social network was crucial for older persons during the lockdown, as some reported feeling cut off from society (22). Communication technology was rapidly identified as a coping strategy in hospitals and nursing homes. Health teams often used the technology of tablets to help older people keep in touch with their families in times when visits were forbidden. Results from the current study extend current knowledge as findings about the role of information technology and internet access in preventing older adults from feeling lonely and socially disconnected are discordant (23–25). We believe that our observation adds further evidence that supports the need to foster the training and supervision of older persons in the use of communication technology to reduce their loneliness and social isolation.

The lack of association between participants' age and their experience of the lockdown was also contrary to our hypothesis. As some studies suggest, the relationship between age and coping mechanisms could be more complex, older people showing more resilience than younger ones during stressful times (12). Similarly, the lack of association between a partner's death in the last 12 months and the lockdown experience could be surprising. However, this

For peer review only - http://bmjopen.bmj.com/site/about/guidelines.xhtml

likely resulted from the low (2.2%) incidence of widowhood in the study population. Unfortunately, we did not have any data on the specific timing and cause of death and were thus unable to investigate the likelihood of an association in a subgroup whose spouse died recently, and/or from a SARS-CoV-2 infection.

Interestingly, this study further contributed to highlighting the independent association between the lockdown experience and several characteristics of participants' living environment. Although none of these characteristics showed a significant independent association in a consistent direction (i.e., a decreased risk of a negative experience and an increased chance of a positive experience or reversely), living in a house and having access to a terrace or a garden appeared to influence the lockdown experience in one of the hypothesized directions. Fortunately, very few participants (5.3%) had no balcony, terrace or garden, a factor that would definitely need examining, should a lockdown be decided again in the future.

The robust observation of the association between participants' fear of falling and their experience of the lockdown is meaningful, too. Indeed, their fear of falling was the only variable that remained independently associated in a consistent direction with their experience of the lockdown. Fearful participants were both at an increased relative risk of reporting a negative experience and at a lower relative risk of reporting a positive experience. Notably, this association remained, even after extensive adjustment for other measurements of the health status (including anxiety), as well as socio-demographic, living environment and social status characteristics. A possible explanation could be that the fear of falling may reflect a state of increased combined mental and physical vulnerability, as also suggested by other studies in community-dwelling older persons that found a robust association with their outdoor mobility and quality of life (26–28). Further research should investigate whether the fear of falling should be addressed through interventions.

For peer review only - http://bmjopen.bmj.com/site/about/guidelines.xhtml

Finally, the observation that dog owners were particularly unlikely to report a negative experience, whereas cat owners were not, deserves a comment. Although this finding may seem futile at first glance, it suggests that the relationship between dog ownership and the experience of the lockdown occurred through enhanced mobility rather than merely through possessing a pet animal. This goes in line with a recent Japanese study (29), which showed an association between dog ownership and a reduced risk of disability, possibly because of the resulting moderate physical activity maintained. Alternative explanations could also be provided by previously reported differences between cat and dog owners in their feeling of loneliness and social isolation, as well as by differences in personality patterns (30,31). To encourage older people to practice dog walking could be an interesting option in times of a lockdown – and probably also in general – as it might prevent them from developing disability and frailty, as well as help them enjoy more social contact and hence reduce their feeling of loneliness.

Strengths and limitations

A major strength of this population-based study is the use of postal – rather than online – questionnaires, for it allowed to include the subgroup of individuals who are uncomfortable with the use of communication technology. As a result, this study reached a more representative sample of community-dwelling older persons. The fact that the questionnaires were completed during the lockdown excludes any memory bias. Furthermore, the design of the study allowed linking data collected during the lockdown with key characteristics routinely assessed in the Lc65+ study.

This study has certain limitations. First, because the results were obtained from community-dwelling adults aged 71 to 86 years, they cannot be generalized to all older individuals. Second, most vulnerable participants had to be excluded from the study due to missing data or

For peer review only - http://bmjopen.bmj.com/site/about/guidelines.xhtml

institutionalization. Fortunately, excluded individuals represented only a very low percentage of our total sample (7.5%). In terms of external validity, this study focused on a specific type of lockdown, i.e., a partial lockdown. Many other European or countries worldwide implemented a stricter or even a total lockdown at the same period. This study also focused on the specific part of the older population who is living in the community, in a relatively good health and who are relatively independent. Accordingly, it might be that the partial lockdown was less restrictive for this category of older persons regarding social interactions, than for older persons living in a nursing home or visiting day-care facilities.

Future implications

This study identified that many participants' characteristics about socio-demography, living environment, health and social status were related with their experience of the lockdown. From a public health perspective, this study further highlights the importance of several specific interventions in reducing the impact of a potential new lockdown in the future. For example, maintaining a higher level of mobility could be achieved by means of interventions such as accompanying older people for outside walks or more specifically by means of actions that alleviates the fear of falling, even if such interventions must be in line with sanitary measures and individuals' security. Modifying the physical environment and promoting activities such as walking a dog could also be recommended to address social isolation. Finally, this study's findings add to previous evidence of the necessary empowerment of older persons, especially of those who express a feeling of loneliness; by learning how to master and use communication technology, to benefit from more social and emotional support, and keep in touch with their relatives.

For peer review only - http://bmjopen.bmj.com/site/about/guidelines.xhtml

Author contributions D.M.-G. drafted the manuscript, S.F. conducted the analyses, YH supervised the work, designed and conducted the data collection as the principal investigator of the Lc65+ study, CB and LSB designed and supervised the work. All authors read, revised and approved the final manuscript.

Acknowledgements We sincerely thank all participants of the Lc65+ study. We are also grateful to the Lc65+ collaborators for their involvement in the data collection, as well as to Prof. Brigitte Santos-Eggimann for her contribution to the conception of the COVID-19 specific questionnaire.

Funding For this research, the authors have not declared a specific grant from any funding agency in the public, commercial or not-for-profit sectors.

Concerning the Lc65+ study, it has been financed from the beginning exclusively by public funds or non-profit organizations. Currently, funding is provided by the Public Health Department of canton Vaud, the Centre for Primary Care and Public Health (Unisanté) and by a research grant from the Loterie Romande (non-profit organization supporting research and social projects).

Conflicts of interest None declared

Ethics approval The Cantonal Human Research Ethical Committee approved the Lausanne Cohort Lc65+ initial study protocol (Protocol (19/04), decision letter: 23 February 2004) as well as successive amendments and the COVID-19 questionnaire for follow-up. Written informed consent was obtained from all subjects.

Data availability statement All data relevant to the study are included in the article or uploaded as supplementary information.

For peer review only

REFERENCES

1. Pfefferbaum B, North CS. Mental Health and the Covid-19 Pandemic. N Engl J Med [Internet]. 2020 Apr 13 [cited 2021 Feb 5]; Available from: https://www.nejm.org/doi/10.1056/NEJMp2008017

2. Tull MT, Edmonds KA, Scamaldo KM, Richmond JR, Rose JP, Gratz KL. Psychological Outcomes Associated with Stay-at-Home Orders and the Perceived Impact of COVID-19 on Daily Life. Psychiatry Res. 2020 Jul;289:113098.

3. Qiu J, Shen B, Zhao M, Wang Z, Xie B, Xu Y. A nationwide survey of psychological distress among Chinese people in the COVID-19 epidemic: implications and policy recommendations. Gen Psychiatry [Internet]. 2020 Mar 6 [cited 2021 Feb 5];33(2). Available from: https://www.ncbi.nlm.nih.gov/pmc/articles/PMC7061893/

4. Gandre C., Coldefy M., Rochereau T. / collab. Les inégalités face au risque de détresse psychologique pendant le confinement : Premiers résultats de l'enquête COCLICO du 3 au 14 avril 2020. Quest Econ SANTE IRDES. 2020 Jun;(249):8p.

5. Wang C, Pan R, Wan X, Tan Y, Xu L, Ho CS, et al. Immediate Psychological Responses and Associated Factors during the Initial Stage of the 2019 Coronavirus Disease (COVID-19) Epidemic among the General Population in China. Int J Environ Res Public Health [Internet]. 2020 Mar [cited 2021 Feb 5];17(5). Available from: https://www.ncbi.nlm.nih.gov/pmc/articles/PMC7084952/

6. Iob E, Frank P, Steptoe A, Fancourt D. Levels of Severity of Depressive Symptoms Among At-Risk Groups in the UK During the COVID-19 Pandemic. JAMA Netw Open. 2020 Oct 1;3(10):e2026064.

7. Girdhar R, Srivastava V, Sethi S. Managing mental health issues among elderly during COVID-19 pandemic. 2020;4.

8. Pierce M, Hope H, Ford T, Hatch S, Hotopf M, John A, et al. Mental health before and during the COVID-19 pandemic: a longitudinal probability sample survey of the UK population. Lancet Psychiatry. 2020 Oct;7(10):883–92.

9. González-Sanguino C, Ausín B, Castellanos MÁ, Saiz J, López-Gómez A, Ugidos C, et al. Mental health consequences during the initial stage of the 2020 Coronavirus pandemic (COVID-19) in Spain. Brain Behav Immun. 2020 Jul;87:172–6.

10. Luo M, Guo L, Yu M, Jiang W, Wang H. The psychological and mental impact of coronavirus disease 2019 (COVID-19) on medical staff and general public - A systematic review and meta-analysis. Psychiatry Res. 2020 Sep;291:113190.

11. Benke C, Autenrieth LK, Asselmann E, Pané-Farré CA. Lockdown, quarantine measures, and social distancing: Associations with depression, anxiety and distress at the beginning of the COVID-19 pandemic among adults from Germany. Psychiatry Res. 2020 Nov;293:113462.

For peer review only - http://bmjopen.bmj.com/site/about/guidelines.xhtml

12. López J, Perez-Rojo G, Noriega C, Carretero I, Velasco C, Martinez-Huertas JA, et al. Psychological well-being among older adults during the COVID-19 outbreak: a comparative study of the young–old and the old–old adults. Int Psychogeriatr. 2020 May 17;1–6.

13. Röhr S, Reininghaus U, Riedel-Heller SG. Mental wellbeing in the German old age population largely unaltered during COVID-19 lockdown: results of a representative survey. BMC Geriatr. 2020 Nov 23;20(1):489.

14. Macdonald B, Hülür G. Well-Being and Loneliness in Swiss Older Adults During the COVID-19 Pandemic: The Role of Social Relationships. The Gerontologist. 2021 Mar 1;61(2):240–50.

15. Falvo I, Zufferey MC, Albanese E, Fadda M. Lived experiences of older adults during the first COVID-19 lockdown: A qualitative study. PLOS ONE. 2021 Jun 23;16(6):e0252101.

16. Henchoz Y, Blanco JM, Fustinoni S, Nanchen D, Büla C, Seematter-Bagnoud L, et al. Cohort Profile: The Lausanne cohort 65+ (Lc65+). Int J Epidemiol. 2021 Nov 29;dyab245.

17. Santos-Eggimann B, Karmaniola A, Seematter-Bagnoud L, Spagnoli J, Büla C, Cornuz J, et al. The Lausanne cohort Lc65+: a population-based prospective study of the manifestations, determinants and outcomes of frailty. BMC Geriatr. 2008 Aug 18;8:20.

18. Lubben J, Blozik E, Gillmann G, Iliffe S, von Renteln Kruse W, Beck JC, et al. Performance of an Abbreviated Version of the Lubben Social Network Scale Among Three European Community-Dwelling Older Adult Populations. The Gerontologist. 2006 Aug;46(4):503–13.

19. Rubin DB. Multiple Imputation for Nonresponse in Surveys. In: Multiple Imputation for Nonresponse in Surveys [Internet]. John Wiley & Sons, Ltd; 1987 [cited 2021 Nov 16]. Available from: https://onlinelibrary.wiley.com/doi/abs/10.1002/9780470316696.fmatter

20. Zaninotto P, Iob E, Demakakos P, Steptoe A. Immediate and Longer-Term Changes in the Mental Health and Well-being of Older Adults in England During the COVID-19 Pandemic. JAMA Psychiatry. 2022 Feb;79(2):1–9.

21. Michael YL, Berkman LF, Colditz GA, Kawachi I. Living Arrangements, Social Integration, and Change in Functional Health Status. Am J Epidemiol. 2001 Jan 15;153(2):123–31.

22. Portacolone E, Chodos A, Halpern J, Covinsky KE, Keiser S, Fung J, et al. The Effects of the COVID-19 Pandemic on the Lived Experience of Diverse Older Adults Living Alone With Cognitive Impairment. Bowers BJ, editor. The Gerontologist. 2021 Feb 23;61(2):251–61.

23. Eghtesadi M. Breaking Social Isolation Amidst COVID-19: A Viewpoint on Improving Access to Technology in Long-Term Care Facilities. J Am Geriatr Soc. 2020 May;68(5):949–50.

24. Noone C, McSharry J, Smalle M, Burns A, Dwan K, Devane D, et al. Video calls for reducing social isolation and loneliness in older people: a rapid review. Cochrane Database Syst Rev. 2020 May 22;2020(5):CD013632.

For peer review only - http://bmjopen.bmj.com/site/about/guidelines.xhtml

25. Bonsaksen T, Thygesen H, Leung J, Ruffolo M, Schoultz M, Price D, et al. Video-Based Communication and Its Association with Loneliness, Mental Health and Quality of Life among Older People during the COVID-19 Outbreak. Int J Environ Res Public Health. 2021 Jun 10;18(12):6284.

26. Schoene D, Heller C, Aung YN, Sieber CC, Kemmler W, Freiberger E. A systematic review on the influence of fear of falling on quality of life in older people: is there a role for falls? Clin Interv Aging. 2019 Apr;Volume 14:701–19.

27. Scheffer AC, Schuurmans MJ, van Dijk N, van der Hooft T, de Rooij SE. Fear of falling: measurement strategy, prevalence, risk factors and consequences among older persons. Age Ageing. 2008 Jan;37(1):19–24.

28. Spaltenstein J, Bula C, Santos-Eggimann B, Krief H, Seematter-Bagnoud L. Factors associated with going outdoors frequently: a cross-sectional study among Swiss community-dwelling older adults. BMJ Open [Internet]. 2020 Aug 24 [cited 2021 Jan 15];10(8). Available from: https://www.ncbi.nlm.nih.gov/pmc/articles/PMC7449269/

29. Taniguchi Y, Seino S, Headey B, Hata T, Ikeuchi T, Abe T, et al. Evidence that dog ownership protects against the onset of disability in an older community-dwelling Japanese population. Mogi M, editor. PLOS ONE. 2022 Feb 23;17(2):e0263791.

30. Hajek A, König HH. How do cat owners, dog owners and individuals without pets differ in terms of psychosocial outcomes among individuals in old age without a partner? Aging Ment Health. 2020 Oct 2;24(10):1613–9.

31. Schulz C, König HH, Hajek A. Differences in Self-Esteem Between Cat Owners, Dog Owners, and Individuals Without Pets. Front Vet Sci. 2020 Sep 2;7:552.

For peer review only

FIGURES' CAPTIONS

Figure 1. Flow chart describing the selection of the study population.

Figure 2. Results from the multivariable multinomial logistic regression analysis investigating participant's characteristics associated with a self-reported positive (green boxes, "not difficult at all") and negative (red triangles, "very or extremely difficult") experience of the lockdown, using participants with neutral ("slightly difficult") experience as reference group

Relative risk ratio (RRR) from the multivariable multinomial logistic regression, CI : confidence interval

*** P<.001, ** P<.01, * P<.05

For peer review only

For peer review only - http://bmjopen.bmj.com/site/about/guidelines.xhtml

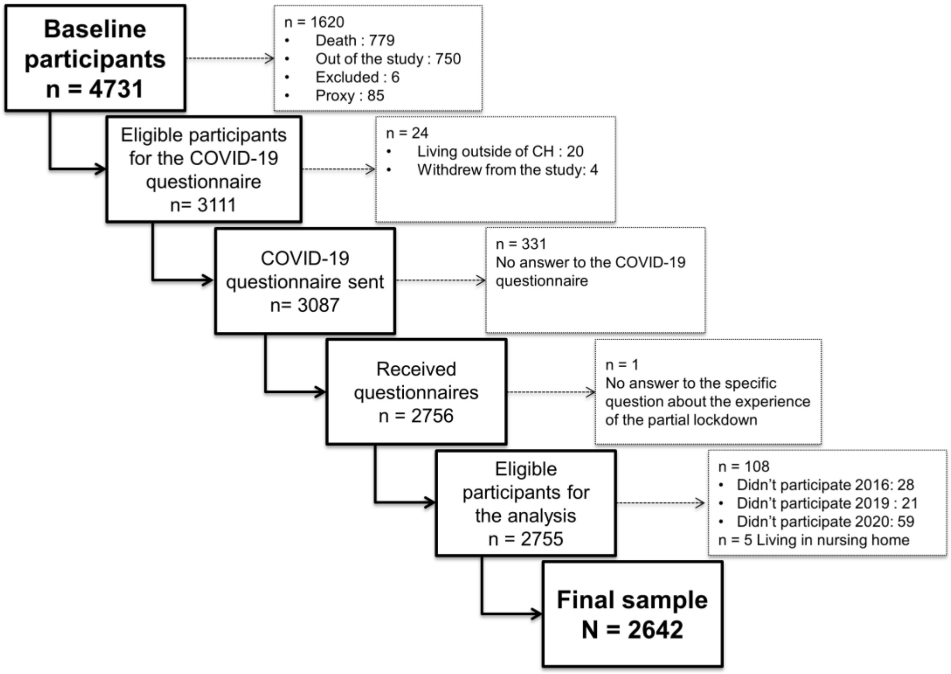

Figure 1. Flow chart describing the selection of the study population.

254x190mm (300 x 300 DPI)

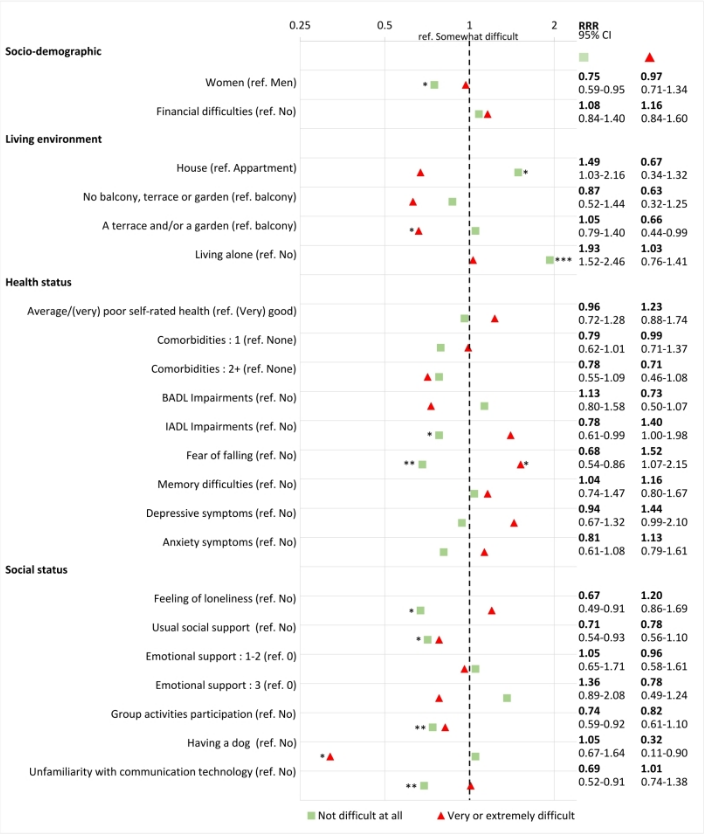

Figure 2. Results from the multivariable multinomial logistic regression analysis investigating participant's characteristics associated with a self-reported positive (green boxes, "not difficult at all") and negative (red triangles, "very or extremely difficult") experience of the lockdown, using participants with neutral ("slightly difficult") experience as reference group

Relative risk ratio (RRR) from the multivariable multinomial logistic regression, CI : confidence interval

*** P<.001, ** P<.01, * P<.05

209x297mm (300 x 300 DPI)

Supplementary material

Supplementary Table 1. Source of study variables in the questionnaires and time of measurement

1. Lubben J, Blozik E, Gillmann G, Iliffe S, von Renteln Kruse W, Beck JC, et al. Performance of an Abbreviated Version of the Lubben Social Network Scale Among Three European Community-Dwelling Older Adult Populations. The Gerontologist. août 2006;46(4):503-13.

**Outcome**	**Measure – Question in the lockdown questionnaire (qlock) April – May 2020**
**Experience of the lockdown**	qlock Q19 "To what extent is the lockdown currently difficult to cope with?"<br>With the choice of answers:<br>1. Not at all difficult<br>2. Slightly difficult<br>3. Very difficult or 4. Extremely difficult (grouped as one category)

**Characteristics**	**Measure – Questions in the yearly questionnaires (q2019, q2020, q2016), the baseline questionnaire (qbase) and lockdown questionnaire (qlock)**
**Age**	Mean (standard deviation)<br>2020 – birth year
**Gender**	Men / Women
**Education level**	qbase Q13 "What is the highest level of education you have completed?"<br>1. Compulsory schooling<br>2. Apprenticeship<br>3. Baccalaureate, high school diploma<br>4. Federal master's degree, professional diploma<br>5. University, college<br>3 groups: 1 Basic compulsory/2 Apprenticeship/3 Post-compulsory schooling
**Frequency of going out of home (homebound status)**	q2019 Q31 (Q30 C2) "How frequently do you go out of your home?"<br>1. 5-7 days per week (non-homebound)<br>2. 3-4 days per week + 3. 1-2 days per week (grouped as one category: semi-homebound)<br>4. I almost never go out of my home (homebound)
**Living situation in 2020:**<br>**1. Living alone**<br>**2. Home type**<br>**3. Home location**<br>**4. Home characteristics**<br>**5. Having a pet animal**	1. qlock Q7 "What are your living arrangements during this lockdown period due to Covid?"<br>2 groups: answer 1 (alone) versus answer 2 (with … other persons)<br>2. qlock Q8a. "What are the characteristics of your living accommodation?"<br>apartment versus house<br>3. qlock Q8b. "What are the characteristics of your living accommodation?"<br>2 groups: in town; suburbs or country

For peer review only

For peer review only - http://bmjopen.bmj.com/site/about/guidelines.xhtml

	4. qlock Q8c.+d. "What are the characteristics of your living accommodation?" balcony (yes/no) + terrace and/or garden (yes/no)<br><br>3 groups: no balcony/terrace/garden; having a balcony; having a terrace or a garden<br><br>5. q2020 Q61 (C2 56) "Do you live with a pet animal?" (yes/no)<br><br>q2020 Q62 (C2 57) "What animal(s) do you live with?"<br>a) cat(s)<br>b) dog(s)<br>c) other(s): specify<br><br>2 variables: Having a cat (yes/no) / Having a dog (yes/no)
**Indicators of low economic status (financial difficulties)**	1. q2019 Q51 (C2 Q45) "Do you currently receive state subsidies for your health insurance costs?" (yes/no)<br>2. q2019 Q52 (C2 Q46) "Do you currently receive complementary subsidies (PC) from the old age and survivor's insurance (AVS)?" (yes/no)<br><br>2 groups: absence of difficulties versus answering yes at one of the 2 questions
**Self-judgement of health status**	q2019, Q1 "In your opinion, what is your current state of health?"<br><br>answer 1+2 (very good and good) vs. 3+4+5 (average, poor, very poor)
**Comorbidities**	q2019 Q4: "During the past 12 months, were you treated, or did you suffer from any of the following health problems, diagnosed by a physician?"<br><br>-No illness (item 20) versus<br>-One of:<br>-Two or more of:<br><br>-Cardiac comorbidities: coronaropathy (3) + cardiac insufficiency (4) + valvular or cardiac muscle deficiency (5)<br>-TIA, stroke (6)<br>-Diabetes (7)<br>-Chronic pulmonary conditions (8) and asthma (9)<br>-Osteoarticular conditions: osteoporosis (10) + fractures (11) + arthrosis or arthritis (12)<br>-Oncologic illness (13)<br>-Parkinson and Alzheimer (16) (17)
**Basic activities of daily living (BADL) and instrumental activities of daily living (IADL)**	q2019 Q43 (C2 Q40) "Do you have difficulties or receive help for the following activities due to physical, psychological, emotional or memory problems?" (bathing, getting dressed, toileting, transferring, feeding, continence (BADL); ability to use the telephone, to use transportations, prepare food, shop for groceries, do the housekeeping, prepare and take your medications, manage finances (IADL))<br><br>2 groups: independent BADL versus difficulties with at least 1 BADL<br>2 groups: independent IADL versus difficulties with at least 1 IADL
**Indicators of mobility:**<br><br>**1. Walking difficulties**<br><br>**2. Fear of falling**	1. q2019 Q35a (C2 Q34a) "Do you find it difficult to walk 100 meters for health reasons?" (yes/no)<br>2. q2019 Q37 (C2 Q36) "Do you fear to fall?" (yes/no)
**Memory difficulties**	q2019 Q5 item 7, 8, 9 "Have you been disrupted since at least 6 months by memory gaps that affect your daily life or by concentration difficulties or decision-making difficulties?" (yes/no)

For peer review only - http://bmjopen.bmj.com/site/about/guidelines.xhtml

**Depressive symptoms (self-reported)**	1. q2019 Q17 "During the past 4 weeks, did you often feel sad, depressed or discouraged?" (yes/no)<br>2. q2019 Q18 "During the past 4 weeks, did you feel a loss of interest or pleasure in your usual activities?" (yes/no)<br><br>2 groups: no symptoms versus presence of symptoms (sadness or apathy)
**Anxiety symptoms (self-reported)**	1. q2019 Q19 "During the past 4 weeks, did you often feel preoccupied and anxious?" (yes/no)<br>2. q2019 Q20 "During the past 4 weeks, how often did you feel calm, balanced, serene?" (rarely, never vs. other responses)<br><br>2 groups: no symptoms versus yes to question Q19 and/or sometimes/rarely/never to question Q20
**Feeling of loneliness**	q2019 Q21 "During the past 4 weeks, how often did you feel isolated?" (always, very often, often, sometimes, rarely, never)<br><br>2 groups: always/very often/often/sometimes versus rarely/never
**Usual social support**	1. q2016 Q44 (C2 Q32) With how many members of your family (partner, children, etc.) do you:<br>a) get in touch (visually or on the phone) at least once a month?<br>b) feel close enough to ask them for help when needed?<br>c) feel at ease to discuss private matters?<br><br>2. q2016 Q45 (C2 Q33) With how many friends or neighbors do you :<br>a) get in touch (visually or on the phone) at least once a month?<br>b) feel close enough to ask them for help when needed?<br>c) feel at ease to discuss private matters?<br><br>2 groups according to clinical cut point of Lubben Social Network Scale (2):<br>Socially supported (LSNS-6≥12) / Not socially supported (LSNS-6<12)
**Emotional Support**	1. q2016 Q46 (C2 Q34) How often can you count on someone to show you love or affection when needed? (always, very often, often, sometimes, rarely, never)<br>2. q2016 Q47 (C2 Q35) How often can you count on someone with whom you can share your intimate preoccupation when needed? (always, very often, often, sometimes, rarely, never)<br>3. q2016 Q48 (C2 Q36) How often can you rely on someone you love and for whom you are important? (always, very often, often, sometimes, rarely, never)<br><br>3 groups: no emotional support declared as always, very often, often; 1-2 emotional support declared as always, very often, often; 3 emotional support declared as always, very often, often
**Familiarity with communication technology**	q2020 Q54 (C1 Q59) "Do you forgo certain activities because you are unfamiliar with technology?" (yes/no)
**Previous participation in group activities**	q2016 Q49 (C2 Q37) How many times a month do you participate in group activities (such as recreation activities, local activities, charitable associations, political or religious groups)<br><br>2 groups: 0 times versus > 0 times
**Death of partner**	q2019 Q56 (C2 Q47) j: "During the past 12 months, did you have to face with the death of your spouse or partner?" (yes/no)

For peer review only - http://bmjopen.bmj.com/site/about/guidelines.xhtml

Supplementary Table 2. Comparisons of characteristics in participants included vs not included in the study

	Total N=2755	Included N=2642	Not included N=113	P-value*
**Age, mean (sd)**	78.1 (4.2)	78.0 (4.2)	79.3 (4.6)	**.002**
**Gender, n (%)**				
Men	1093 (39.7)	1059 (40.1)	34 (30.1)	**.033**
Women	1662 (60.3)	1583 (59.9)	79 (69.9)	
**Education level, n (%)**				
Basic compulsory	438 (15.9)	404 (15.3)	34 (30.1)	**<.001**
Apprenticeship	1072 (39.0)	1032 (39.1)	40 (35.4)	
Post-compulsory schooling	1242 (45.1)	1203 (45.6)	39 (34.5)	
**Depressive symptoms, n (%)**				
No	1962 (72.0)	1916 (72.6)	46 (53.5)	**<.001**
Yes	763 (28.0)	723 (27.4)	40 (46.5)	
**Self-rated health, n (%)**				
Very good / good	1775 (65.1)	1734 (65.6)	41 (47.7)	**.001**
Average / poor / very poor	953 (34.9)	908 (34.4)	45 (52.3)	
**Living alone, n (%)**				
No	1527 (55.6)	1465 (55.6)	62 (55.4)	.956
Yes	1219 (44.4)	1169 (44.4)	50 (44.6)	
**Exp. of the lockdown, n (%)**				
Positive	596 (21.6)	569 (21.5)	27 (23.9)	
Neutral	1854 (67.3)	1791 (67.8)	63 (55.8)	
Negative	305 (11.1)	282 (10.7)	23 (20.3)	**.003**

*P-values from Student's t-test for age and from Pearson's chi2 test for categorical variables.

For peer review only

Supplementary Table 3. Results of multivariable multinomial logistic regression investigating participants' characteristics associated with a positive ("not at all difficult") and negative ("very or extremely difficult") experience of the lockdown, using participants with a neutral ("slightly difficult") experience as reference group

	**Experience of the lockdown**					
	Positive (ref. neutral)			Negative (ref. neutral)		
	RRR	95% CI	P-value*	RRR	95% CI	P-value*
Socio-demographic						
**Gender**						
Men	ref.			ref.		
Women	**0.75**	**0.59 – 0.95**	**.016**	0.97	0.71 – 1.34	.873
**Financial difficulties**						
No	ref.			ref.		
Yes	1.08	0.84 – 1.40	.555	1.16	0.84 – 1.60	.353
Living environment						
**Living alone**						
No	ref.			ref.		
Yes	**1.93**	**1.52 – 2.46**	**<.001**	1.03	0.76 – 1.41	.836
**Home type**						
Apartment	ref.			ref.		
House	**1.49**	**1.03 – 2.16**	**.033**	0.67	0.34 – 1.32	.247
**Home characteristics**						
No balcony, terrace or garden	0.87	0.52 – 1.44	.578	0.63	0.32 – 1.25	.186
A balcony	ref.			ref.		
A terrace and/or a garden	1.05	0.79 – 1.40	.738	**0.66**	**0.44 – 0.99**	**.044**
Health status						
**Self-rated health**						
Very good / good	ref.			ref.		
Average / poor/ very poor	0.96	0.72 – 1.28	.761	1.23	0.88 – 1.74	.231
**Comorbidities**						
None	ref.			ref.		
1	0.79	0.62 – 1.01	.061	0.99	0.71 – 1.37	.952
2+	0.78	0.55 – 1.09	.144	0.71	0.46 – 1.08	.107
**BADL impairment**						
Independent	ref.			ref.		
Diff. / help for at least 1	1.13	0.80 – 1.58	.493	0.73	0.50 – 1.07	.104
**IADL impairment**						
Independent	ref.			ref.		
Diff. / help for at least 1	**0.78**	**0.61 – 0.99**	**.045**	1.40	1.00 – 1.98	.053
**Fear of falling**						
No	ref.			ref.		
Yes	**0.68**	**0.54 – 0.86**	**.001**	**1.52**	**1.07 – 2.15**	**.019**
**Memory difficulties**						
No	ref.			ref.		
Yes	1.04	0.74 – 1.47	.816	1.16	0.80 – 1.67	.440

For peer review only

**Depressive symptoms**						
No	ref.			ref.		
Yes	0.94	0.67 – 1.32	.737	1.44	0.99 – 2.10	.059
**Anxiety symptoms**						
No	ref.			ref.		
Yes	0.81	0.61 – 1.08	.151	1.13	0.79 – 1.61	.511
Social status						
**Feeling of loneliness**						
No	ref.			ref.		
Yes	**0.67**	**0.49 – 0.91**	**.011**	1.20	0.86 – 1.69	.281
**Socially supported**						
No	ref.			ref.		
Yes	**0.71**	**0.54 – 0.93**	**.012**	0.78	0.56 – 1.10	.158
**Emotional support**						
0	ref.			ref.		
1-2	1.05	0.65 – 1.71	.842	0.96	0.58 – 1.61	.889
3	1.36	0.89 – 2.08	.158	0.78	0.49 – 1.24	.293
**Previous participation in group activities**						
No	ref.			ref.		
Yes	**0.74**	**0.59 – 0.92**	**.007**	0.82	0.61 – 1.10	.193
**Having a dog**						
No	ref.			ref.		
Yes	1.05	0.67 – 1.64	.848	**0.32**	**0.11 – 0.90**	**.031**
**Unfamiliarity with technology**						
No	ref.			ref.		
Yes	**0.69**	**0.52 – 0.91**	**.008**	1.01	0.74 – 1.38	.952

Choice of variables: significant variables (threshold p<.05) in the bivariable multinomial logistic regression

Notes: N=2,246 complete cases (outcome distributed into 470/1,540/236)

RRR = relative risk ratio; CI = confidence interval. Reference: "slightly difficult"

* P-values from adjusted multinomial logistic regression.

For peer review only

For peer review only - http://bmjopen.bmj.com/site/about/guidelines.xhtml

Supplementary Table 3bis. Results from the multivariable multinomial logistic regression after multivariable imputation for missing data (sensitivity analysis).

	Experience of the lockdown					
	Not at all difficult (ref. slightly difficult)			Very or extremely difficult (ref. slightly difficult)		
	RRR	95% CI	P-value*	RRR	95% CI	P-value*
Socio-demographic						
**Gender**						
Men	ref.			ref.		
Women	**0.70**	**0.56 – 0.87**	**.001**	1.03	0.76 – 1.38	.858
**Financial difficulties**						
No	ref.			ref.		
Yes	1.10	0.87 – 1.38	.437	1.15	0.87 – 1.54	.326
Living environment						
**Living alone**						
No	ref.			ref.		
Yes	**1.90**	**1.52 – 2.37**	**<.001**	1.10	0.83 – 1.46	.519
**Home type**						
Apartment	ref.			ref.		
House	1.30	0.92 – 1.83	.131	0.70	0.37 – 1.32	.276
**Home characteristics**						
No balcony, terrace or garden	0.89	0.56 – 1.39	.596	0.62	0.33 – 1.13	.119
A balcony	ref.			ref.		
A terrace and/or a garden	1.10	0.85 – 1.42	.473	**0.62**	**0.43 – 0.90**	**.012**
Health status						
**Self-rated health**						
Very good / good	ref.			ref.		
Average / poor / very poor	0.99	0.77 – 1.29	.966	1.23	0.90 – 1.69	.198
**Comorbidities**						
None	ref.			ref.		
1	0.83	0.66 – 1.03	.094	1.03	0.76 – 1.40	.838
2+	0.89	0.65 – 1.21	.442	0.83	0.56 – 1.21	.325
**BADL impairment**						
Independent	ref.			ref.		
Diff. / help for at least 1	1.06	0.78 – 1.44	.713	0.77	0.55 – 1.10	.150
**IADL impairment**						
Independent	ref.			ref.		
Diff. / help for at least 1	**0.75**	**0.60 – 0.95**	**.016**	1.21	0.89 – 1.66	.227
**Fear of falling**						
No	ref.			ref.		
Yes	**0.73**	**0.59 – 0.90**	**.004**	**1.43**	**1.04 – 1.96**	**.029**
**Memory difficulties**						
No	ref.			ref.		
Yes	1.15	0.85 – 1.56	.366	1.09	0.77 – 1.53	.630
**Depressive symptoms**	ref.					
No	1.01	0.75 – 1.37		ref.	**1.16 – 2.31**	

For peer review only - http://bmjopen.bmj.com/site/about/guidelines.xhtml

Yes			.950	**1.63**		**.005**
**Anxiety symptoms**						
No	ref.			ref.		
Yes	0.78	0.60 – 1.01	.062	1.04	0.75 – 1.44	.829
Social status						
**Feeling of loneliness**						
No	ref.			ref.		
Yes	**0.62**	**0.47 – 0.83**	**.001**	1.07	0.78 – 1.46	.678
**Usual social support**						
No	ref.			ref.		
Yes	**0.73**	**0.57 – 0.94**	**.014**	0.80	0.58 – 1.12	.191
**Emotional support**						
0	ref.			ref.		
1-2	0.91	0.59 – 1.39	.651	0.94	0.59 – 1.50	.798
3	1.09	0.76 – 1.57	.646	0.77	0.51 – 1.18	.230
**Previous participation in group activities**						
No	ref.			ref.		
Yes	**0.79**	**0.65 – 0.97**	**.024**	0.83	0.64 – 1.09	.192
**Having a dog**						
No	ref.			ref.		
Yes	1.04	0.69 – 1.58	.849	**0.29**	**0.10 – 0.79**	**.016**
**Unfamiliarity with communication technology**						
No	ref.			ref.		
Yes	**0.66**	**0.52 – 0.85**	**.001**	1.12	0.85 – 1.49	.422

RRR relative risk ratio; CI confidence interval.

Reference: “slightly difficult” lockdown’s experience

* P-values from adjusted multinomial logistic regression.

For peer review only

For peer review only - http://bmjopen.bmj.com/site/about/guidelines.xhtml

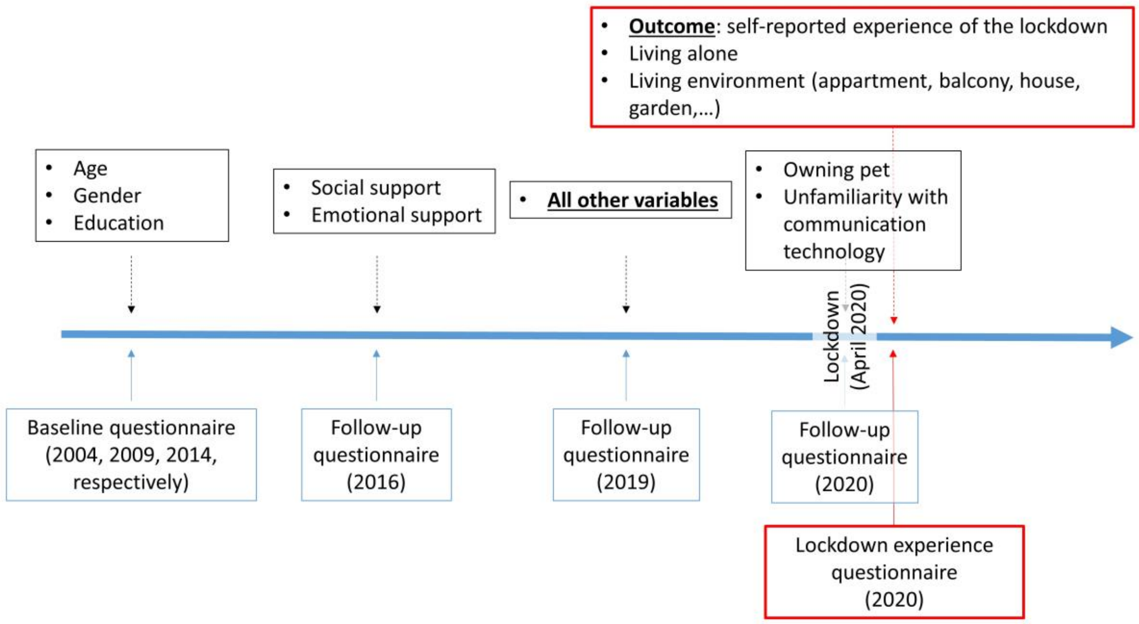

Supplementary Figure 1. Description of the sources of the study's variables and outcome

For peer review only

STROBE 2007 (v4) Statement—Checklist of items that should be included in reports of *cohort studies*

Section/Topic	Item #	Recommendation	Reported on page #
**Title and abstract**	1	(*a*) Indicate the study's design with a commonly used term in the title or the abstract	2
		(*b*) Provide in the abstract an informative and balanced summary of what was done and what was found	2
**Introduction**			
Background/rationale	2	Explain the scientific background and rationale for the investigation being reported	5,6
Objectives	3	State specific objectives, including any prespecified hypotheses	6,7
**Methods**			
Study design	4	Present key elements of study design early in the paper	8
Setting	5	Describe the setting, locations, and relevant dates, including periods of recruitment, exposure, follow-up, and data collection	8,10 + Supplementary table 4 + Supplementary Figure 1
Participants	6	(*a*) Give the eligibility criteria, and the sources and methods of selection of participants. Describe methods of follow-up	8
		(*b*) For matched studies, give matching criteria and number of exposed and unexposed	Not applicable
Variables	7	Clearly define all outcomes, exposures, predictors, potential confounders, and effect modifiers. Give diagnostic criteria, if applicable	8-9
Data sources/ measurement	8*	For each variable of interest, give sources of data and details of methods of assessment (measurement). Describe comparability of assessment methods if there is more than one group	9, Supplementary table 4 + Supplementary Figure 1
Bias	9	Describe any efforts to address potential sources of bias	8
Study size	10	Explain how the study size was arrived at	12 (In results section) + Supplementary Figure 1
Quantitative variables	11	Explain how quantitative variables were handled in the analyses. If applicable, describe which groupings were chosen and	Not applicable

For peer review only

		why	
Statistical methods	12	(*a*) Describe all statistical methods, including those used to control for confounding	10
		(*b*) Describe any methods used to examine subgroups and interactions	10
		(*c*) Explain how missing data were addressed	10
		(*d*) If applicable, explain how loss to follow-up was addressed	Not applicable
		(*e*) Describe any sensitivity analyses	10
**Results**			
Participants	13*	(a) Report numbers of individuals at each stage of study—eg numbers potentially eligible, examined for eligibility, confirmed eligible, included in the study, completing follow-up, and analysed	12
		(b) Give reasons for non-participation at each stage	12
		(c) Consider use of a flow diagram	Figure 1
Descriptive data	14*	(a) Give characteristics of study participants (eg demographic, clinical, social) and information on exposures and potential confounders	12, 15, 16, Table 1
		(b) Indicate number of participants with missing data for each variable of interest	Table 1 (notes)
		(c) Summarise follow-up time (eg, average and total amount)	Not applicable
Outcome data	15*	Report numbers of outcome events or summary measures over time	Table 1 (experience of the lockdown)
Main results	16	(*a*) Give unadjusted estimates and, if applicable, confounder-adjusted estimates and their precision (eg, 95% confidence interval). Make clear which confounders were adjusted for and why they were included	16, 18-20, Table 1, 2, Figure 2
		(*b*) Report category boundaries when continuous variables were categorized	Not applicable
		(*c*) If relevant, consider translating estimates of relative risk into absolute risk for a meaningful time period	Not applicable
Other analyses	17	Report other analyses done—eg analyses of subgroups and interactions, and sensitivity analyses	20
**Discussion**			
Key results	18	Summarise key results with reference to study objectives	21-24
**Limitations**			
Interpretation	20	Give a cautious overall interpretation of results considering objectives, limitations, multiplicity of analyses, results from similar studies, and other relevant evidence	21-25
Generalisability	21	Discuss the generalisability (external validity) of the study results	25
**Other information**			
Funding	22	Give the source of funding and the role of the funders for the present study and, if applicable, for the original study on	26

For peer review only

		which the present article is based	

*Give information separately for cases and controls in case-control studies and, if applicable, for exposed and unexposed groups in cohort and cross-sectional studies.

Note: An Explanation and Elaboration article discusses each checklist item and gives methodological background and published examples of transparent reporting. The STROBE checklist is best used in conjunction with this article (freely available on the Web sites of PLoS Medicine at http://www.plosmedicine.org/, Annals of Internal Medicine at http://www.annals.org/, and Epidemiology at http://www.epidem.com/). Information on the STROBE Initiative is available at www.strobe-statement.org.

For peer review only

BMJ Open

« Older people's experience of the partial lockdown during the COVID-19 pandemic in Switzerland: a cross-sectional study»

Journal:	*BMJ Open*
Manuscript ID	bmjopen-2022-067167.R1
Article Type:	Original research
Date Submitted by the Author:	02-Feb-2023
Complete List of Authors:	Märki-Germann, Daphné; Lausanne University Hospital Department of Geriatrics and Geriatric Rehabilitation<br>Seematter-Bagnoud , Laurence; Center for Primary Care and Public Health, Epidemiology and Health systems; University Hospital of Lausanne, Geriatric Medicine and Rehabilitation<br>FUSTINONI, Sarah; Center for Primary Care and Public Health, Epidemiology and Health systems<br>SPALTENSTEIN, Julia; Lausanne University Hospital Department of Geriatrics and Geriatric Rehabilitation; Center for Primary Care and Public Health, Epidemiology and Health systems<br>Bula, Christophe; Lausanne University Hospital Department of Geriatrics and Geriatric Rehabilitation<br>HENCHOZ, Yves; Center for Primary Care and Public Health, Epidemiology and Health systems
**Primary Subject Heading**:	Geriatric medicine
Secondary Subject Heading:	Epidemiology, Public health
Keywords:	COVID-19, GERIATRIC MEDICINE, MENTAL HEALTH

SCHOLARONE™
Manuscripts

I, the Submitting Author has the right to grant and does grant on behalf of all authors of the Work (as defined in the below author licence), an exclusive licence and/or a non-exclusive licence for contributions from authors who are: i) UK Crown employees; ii) where BMJ has agreed a CC-BY licence shall apply, and/or iii) in accordance with the terms applicable for US Federal Government officers or employees acting as part of their official duties; on a worldwide, perpetual, irrevocable, royalty-free basis to BMJ Publishing Group Ltd ("BMJ") its licensees and where the relevant Journal is co-owned by BMJ to the co-owners of the Journal, to publish the Work in this journal and any other BMJ products and to exploit all rights, as set out in our licence.

The Submitting Author accepts and understands that any supply made under these terms is made by BMJ to the Submitting Author unless you are acting as an employee on behalf of your employer or a postgraduate student of an affiliated institution which is paying any applicable article publishing charge ("APC") for Open Access articles. Where the Submitting Author wishes to make the Work available on an Open Access basis (and intends to pay the relevant APC), the terms of reuse of such Open Access shall be governed by a Creative Commons licence – details of these licences and which Creative Commons licence will apply to this Work are set out in our licence referred to above.

Other than as permitted in any relevant BMJ Author's Self Archiving Policies, I confirm this Work has not been accepted for publication elsewhere, is not being considered for publication elsewhere and does not duplicate material already published. I confirm all authors consent to publication of this Work and authorise the granting of this licence.

For peer review only

« Older people's experience of the partial lockdown during the COVID-19 pandemic in Switzerland: a cross-sectional study»

Daphné MÄRKI-GERMANN [1]

Laurence SEEMATTER-BAGNOUD, MD, MPH, PhD [1,2]

Sarah FUSTINONI, BSc [2]

Julia SPALTENSTEIN, MD [1,2]

Christophe BÜLA, MD [1]

Yves HENCHOZ, PhD[2]

(1) Service of Geriatric medicine and geriatric rehabilitation, Lausanne University Hospital, Lausanne, Switzerland

(2) Center for Primary Care and Public Health (Unisanté), University of Lausanne, Lausanne, Switzerland

Corresponding author:

Dr Daphné MÄRKI-GERMANN

Lausanne University Hospital (CHUV), Department of Geriatrics and Geriatric Rehabilitation, Chemin de Mont-Paisible 16, 1011 Lausanne

daphne.marki-germann@unil.ch

Word count: 4169

For peer review only

For peer review only - http://bmjopen.bmj.com/site/about/guidelines.xhtml

ABSTRACT

Objectives This study aimed to evaluate older people's experience of a COVID-19 partial lockdown (March 16 - May 11, 2020) in Lausanne, Switzerland.

Setting and participants Community-dwelling participants of the Lausanne cohort Lc65+ in 2020, aged 71–86 years (n=2642).

Design and outcome This cross-sectional study was nested within the Lc65+ longitudinal study. A specific COVID-19 questionnaire was sent on April 17, 2020, to evaluate participants' experience of the lockdown (outcome). Multinomial logistic regression models were used to determine the socio-demographic, living environment, health, and social factors associated.

Results Out of 2,642 participants, 67.8% described the lockdown as "somewhat" difficult (reference group), 21.5% as "not at all" difficult (positive), 10.7% as "very or extremely" difficult (negative). The relative risk of a positive experience was higher in participants living alone (RRR=1.93, [95%CI] 1.52–2.46) or in a house (RRR=1.49, 1.03–2.16); lower in those who reported fear of falling (RRR=0.68, 0.54–0.86), functional difficulties (RRR=0.78, 0.61–0.99), feeling of loneliness (RRR=0.67, 0.49–0.91), unfamiliarity with communication technologies (RRR=0.69, 0.52–0.91), usual social support (RRR=0.71, 0.50–0.93), previous participation in group activities (RRR=0.74, 0.59–0.92) and among women (RRR=0.75, 0.59–0.95). The relative risk of a negative experience was higher in participants with fear of falling (RRR=1.52, 1.07–2.15), and lower in those who had a terrace/garden (RRR=0.66, 0.44–0.99) and owned a dog (RRR=0.32, 0.11–0.90).

For peer review only - http://bmjopen.bmj.com/site/about/guidelines.xhtml

Conclusions Only one in ten participants experienced the lockdown as very or extremely difficult. Specific interventions targeting vulnerability factors, such as fear of falling, could lessen the impact of any future similar situation.

Key words: COVID-19, partial lockdown, risk factors, sociodemographic factors, living environment

For peer review only

ARTICLE SUMMARY

Strengths and limitations of this study

- This study focused on the experience of the COVID-19 partial lockdown in a large sample of community-dwelling older people.
- The design of the study allowed linking data collected during the lockdown with key characteristics routinely assessed in the Lc65+ longitudinal study, and to highlight the ones that were independently associated with a positive and a negative experience of the lockdown.
- Responses were obtained from postal – rather than online – questionnaires, allowing to include individuals who are uncomfortable with the use of communication technology.
- The most vulnerable participants (7.5% of the total sample) had to be excluded from the study due to missing data or institutionalisation.
- Most data stem from the 2019 questionnaire, and we cannot formally exclude minor changes in some participants' situation in 2020.

For peer review only

INTRODUCTION

In Switzerland, the Federal Council ordered a partial lockdown period lasting from March 16 to May 11, 2020 to prevent the spread of the highly contagious SARS-CoV-2 virus. Strong recommendations to stay strictly at home were specifically directed toward older people because of their increased vulnerability to severe COVID-19 infection and hospitalisation.

Concerns rapidly emerged regarding the psychological impact of the lockdown. Early studies conducted during the first outbreak in China and the United States showed increased anxiety and depressive symptoms in the context of stay-at-home orders (1–3). Overall, nearly half the general population reported moderate to severe levels of distress during the first epidemic outbreak (3–6). These early studies suggested that people aged 60 years and over were particularly at risk of such adverse psychological impact (3).

The partial lockdown deprived socially active older people of their usual contact with society. Older people who already were less active because of health problems also faced the fear of being infected through contacts with in-home care staff (7).

Several later studies conducted in other countries reported information about the early impact of the COVID-19 pandemic on anxiety and depressive symptoms in the general population (8–10). Results showed a significantly increasing prevalence of mental distress during the first months of the epidemic, specifically of anxio-depressive and post-traumatic stress disorder symptoms.

Other studies investigated the socio-demographic and health predictors of depression and anxiety symptoms associated with the epidemic and the stay-at-home orders in the general population (10,11). However, few specifically focused on the older population and none on the specific conditions of a partial lockdown. For instance, it remains unclear whether age itself is

For peer review only - http://bmjopen.bmj.com/site/about/guidelines.xhtml

a risk factor for a negative experience or, reversely, whether it could be a protective factor due to resilience and accommodative strategies (12,13). Similarly, the potential mediating effect of mobility and functional capacities has not been investigated.

A study conducted in Zürich, Switzerland, on 99 people aged 65 years and over showed that their level of well-being decreased as their feeling of loneliness increased during the first 4 weeks of the partial lockdown (14). Another Swiss study conducted in the Ticino Canton on 19 community-dwelling older people revealed the latter's ambivalent feelings about the lockdown; some of them expressed their feeling of exclusion because older people were categorised as vulnerable (15).

This study primarily aimed to evaluate how a large sample of older people experienced the partial lockdown during the first wave of the SARS-CoV-2 epidemic in Switzerland. Since social distancing measures particularly targeted the older population, but the lockdown was less strict in Switzerland than in many other countries, our hypothesis was that most participants would report a neutral experience, judging this period as somewhat difficult, whereas two smaller subgroups would report more extreme feelings and consider their experience as either negative (judging the period as very difficult) or positive (judging the period as not difficult at all).

A second aim was to study participants' characteristics associated with these more extreme feelings about their experience of the partial lockdown. More specifically, this study sought to investigate the association of four categories of participants' characteristics (socio-demographic, living environment, health status and social status) with a positive, respectively negative, experience of the lockdown.

We hypothesised an association between vulnerability factors (older age, lower education, higher comorbidities, as well as functional, cognitive, and affective impairments) and a higher

For peer review only - http://bmjopen.bmj.com/site/about/guidelines.xhtml

risk of a negative experience. By contrast, we hypothesised that participants who lived in a more comfortable environment (in a house, with access to a terrace/garden) would be more likely to report a positive experience. Finally, our hypotheses were mixed regarding social factors, as we expected that participants having less usual social support would be more likely to report a negative experience, whereas those owning pet animals might report a positive experience.

For peer review only

For peer review only - http://bmjopen.bmj.com/site/about/guidelines.xhtml

METHODS

Study design and participants

This study is a cross-sectional analysis nested within the Lausanne cohort 65+ (Lc65+) longitudinal study (16,17), initially designed to investigate the development of age-related frailty. Three samples of non-institutionalised individuals living in Lausanne (Switzerland) were successively enrolled in Lc65+ at the same age of 65 to 70 years in 2004 (birth years 1934–1938), 2009 (1939–1943), and 2014 (1944–1948).

For this cross-sectional analysis, data on participants' characteristics stem from the Lc65+ recruitment (2004, 2009, and 2014, respectively) and yearly follow-up (2016, 2019, and 2020) questionnaires. Specific details regarding the process of Lc65+ participants enrollment are available on the original study articles (16,17).

A separate COVID-19 specific questionnaire was sent by postal mail to 3087 Lc65+ eligible participants on April 17, 2020 (details available on Figure 1 for participants' selection). The questionnaires relating to their experience of the partial lockdown were completed during the lockdown to exclude memory bias. As a simplification measure, the "partial lockdown" will henceforth be referred to as "lockdown".

The Lc65+ study protocol, including the COVID-19 specific questionnaire, was approved by the ethical committee for human's research in the canton of Vaud (Protocol No. 19/04). Written informed consent was obtained from the participants.

Measurement of self-reported experience of the lockdown (outcome)

For peer review only - http://bmjopen.bmj.com/site/about/guidelines.xhtml

The study's outcome derived from participants' answer to the following question in the COVID-19 specific questionnaire: "To what extent is the lockdown currently difficult to live with?"

According to their answers, the participants' experience of the lockdown was defined as a) **Positive** in participants who answered "not difficult at all", b) **Neutral** (reference group) in participants who answered "somewhat difficult", and c) **Negative** in those who answered "very difficult" or "extremely difficult". Thus, the term "positive" refers to participants who reported a more positive experience of the lockdown than other participants and does not imply that the lockdown was a positive experience.

Measurement of participants' characteristics

Variables with hypothesised associations with participants' experience of the lockdown (outcome) were identified in the four following domains:

Socio-demographic variables included age (at the end of year 2020), gender, education level (low: compulsory school; medium: apprenticeship; high: college, university degree or equivalent), and financial difficulties (entitlement to supplementary benefits to old-age insurance).

Living environment variables encompassed living alone versus with other persons, home type (apartment versus house), home characteristics (having a balcony, a terrace/garden, or none of the above), home location (countryside/suburbs versus urban area).

Health status variables included self-rated health, the presence or absence of comorbidities, impairment in basic and instrumental activities of daily living (BADL (18), IADL (19)),

For peer review only - http://bmjopen.bmj.com/site/about/guidelines.xhtml

mobility indicators (walking difficulties, fear of falling (20)), reporting memory difficulties or depressive/anxiety symptoms (21,22), and homebound status.

Social status variables included usual social support (23), emotional support (24), feeling of loneliness, the recent death of a partner, being familiar with communication technology, participating in group activities, and owning a dog and/or a cat.

Most information was retrieved from the 2019 questionnaire. If not available that year, data from the latest follow-up was used instead (for more details on timing of data collection for each variable, see Supplementary – Table 1, Supplementary – Figure 1).

Statistical analysis

Proportions of participants reporting a positive, neutral, and negative self-reported experience of the lockdown, respectively, were first compared in bivariable analyses within each characteristic of the study population, using parametric and non-parametric tests (i.e., Chi-square and Fisher exact tests) according to the variables' distribution. Multinomial logistic regression analyses were then performed to investigate the bivariable and multivariable associations between participants' characteristics and a positive or negative experience of the lockdown, using participants with neutral experience as the reference group. Variables that proved statistically significant ($p<.05$) in bivariable analysis were included in the multivariable model.

Interactions were tested between gender and several specific characteristics (fear of falling, living alone, depressive and anxiety symptoms), as well as between living alone and usual social support, instrumental activities of daily living, self-rated health, emotional support, or unfamiliarity with communication technologies.

For peer review only - http://bmjopen.bmj.com/site/about/guidelines.xhtml

To assess the effect of missing data, a sensitivity analysis was performed in a multivariate imputation approach. Assuming that covariables were missing at random, missing values were imputed iteratively by means of chained equations that generated fifty complete datasets. A multivariable analysis was run on each multiply imputed dataset, which produced fifty sets of coefficients and standard errors. The estimates were then pooled according to Rubin's combination rules (25).

Patient and public involvement Patients and/or the public were not involved in the design, conduct, reporting, nor in the dissemination plans of this research.

For peer review only

For peer review only - http://bmjopen.bmj.com/site/about/guidelines.xhtml

RESULTS

Overall, 2,756 of the 3,087 (response rate 89.3%) contacted participants returned their completed COVID-19 questionnaires and 2,755 replied to the question about their experience of the lockdown. Figure 1 details the process of the population selection. After the exclusion of institutionalised participants (n=5), and of those who did not complete the previous questionnaires (n=108), analyses were conducted on 2,642 participants (85.6% of sent questionnaires).

Compared to included participants (Supplementary – Table 2), those excluded (n=113) were older (P=.002), more often women (P=.033), had a lower education level (P<.001), more frequently reported depressive symptoms (P<.001), poor self-rated health (P=.001), and a "very difficult" or "extremely difficult" experience of the lockdown (P=.003).

Table 1 details participants' characteristics and provides the results of the comparisons across study groups according to their experience of the lockdown. Overall, 1,791 (67.8%) participants reported a "somewhat difficult" (neutral, reference) experience of the lockdown, 569 (21.5%) participants reported this experience as "not at all difficult" (positive) and 282 (10.7%) as "very or extremely difficult" (negative).

Table 1. Characteristics of the study population and distributions by positive, neutral, or negative experience of the lockdown.

	Experience of the lockdown*				
	Total population N = 2642	Positive n = 569 (21.5%)	Neutral (reference) n = 1791 (67.8%)	Negative n = 282 (10.7%)	P-value†
Socio-demographic					
**Age, mean (sd)**	78.0 (4.2)	77.8 (4.2)	78.1 (4.1)	78.1 (4.4)	.434

For peer review only - http://bmjopen.bmj.com/site/about/guidelines.xhtml

**Gender, n (%)**					
Men	1059 (100.0)	272 (25.7)	694 (65.5)	93 (8.8)	**<.001**
Women	1583 (100.0)	297 (18.8)	1097 (69.3)	189 (11.9)	
**Education level, n (%)**					
Basic compulsory	404 (100.0)	89 (22.0)	261 (64.6)	54 (13.4)	.245
Apprenticeship	1032 (100.0)	210 (20.4)	712 (69.0)	110 (10.7)	
Post-compulsory schooling	1203 (100.0)	268 (22.3)	817 (67.9)	118 (9.8)	
**Financial difficulties, n (%)**					
No	1960 (100.0)	420 (21.4)	1348 (68.8)	192 (9.8)	**.035**
Yes	681 (100.0)	149 (21.9)	442 (64.9)	90 (13.2)	
Living environment					
**Living alone, n (%)**					
No	1465 (100.0)	288 (19.7)	1037 (70.8)	140 (9.6)	**.001**
Yes	1169 (100.0)	279 (23.9)	748 (64.0)	142 (12.1)	
**Home type, n (%)**					
Apartment	2298 (100.0)	475 (20.7)	1556 (67.7)	267 (11.6)	**<.001**
House	338 (100.0)	91 (26.9)	232 (68.6)	15 (4.4)	
**Home location, n (%)**					
City	2061 (100.0)	428 (20.8)	1401 (68.0)	232 (11.3)	.052
Suburbs/rural areas	559 (100.0)	134 (24.0)	379 (67.8)	46 (8.2)	
**Home characteristics, n (%)**					
No balcony / terrace / garden	140 (100.0)	29 (20.7)	98 (70.0)	13 (9.3)	**<.001**
A balcony	1684 (100.0)	339 (20.1)	1128 (67.0)	217 (12.9)	
A terrace and/or garden	813 (100.0)	198 (24.4)	563 (69.3)	52 (6.4)	
Health status					
**Self-rated health, n (%)**					
Very good / good	1734 (100.0)	419 (24.2)	1173 (67.6)	142 (8.2)	**<.001**
Average / poor / very poor	908 (100.0)	150 (16.5)	618 (68.1)	140 (15.4)	
**Comorbidities, n (%)**					
None	1150 (100.0)	288 (25.0)	760 (66.1)	102 (8.9)	**.001**
1	981 (100.0)	189 (19.3)	675 (68.8)	117 (11.9)	
2+	498 (100.0)	87 (17.5)	350 (70.3)	61 (12.3)	
**BADL impairment‡, n (%)**					
Independent	2172 (100.0)	486 (22.4)	1467 (67.5)	219 (10.1)	**.013**
Difficulty or help for at least 1	464 (100.0)	80 (17.2)	322 (69.4)	62 (13.4)	
**IADL impairment ǁ, n (%)**					
Independent	1239 (100.0)	329 (26.6)	819 (66.1)	91 (7.3)	**<.001**
Difficulty or help for at least 1	1367 (100.0)	225 (16.5)	954 (69.8)	188 (13.8)	
**Walking difficulty, n (%)**					
No	2285 (100.0)	493 (21.6)	1554 (68.0)	238 (10.4)	.546
Yes	339 (100.0)	71 (20.9)	226 (66.7)	42 (12.4)	
**Fear of falling, n (%)**					
No	1136 (100.0)	316 (27.8)	744 (65.5)	76 (6.7)	**<.001**
Yes	1502 (100.0)	253 (16.8)	1044 (69.5)	205 (13.7)	
**Homebound status, n (%)**					
Non-homebound	2046 (100.0)	437 (21.4)	1387 (67.8)	222 (10.9)	.552
Semi-homebound	577 (100.0)	125 (21.7)	394 (68.3)	58 (10.1)	
Homebound	19 (100.0)	7 (36.8)	10 (52.6)	2 (10.5)	

For peer review only

For peer review only - http://bmjopen.bmj.com/site/about/guidelines.xhtml

**Memory difficulties, n (%)**						
	No	2218 (100.0)	492 (22.2)	1507 (67.9)	229 (9.9)	**.002**
	Yes	412 (100.0)	74 (18.0)	275 (66.7)	63 (15.3)	
**Depressive symptoms, n (%)**						
	No	1916 (100.0)	460 (24.0)	1302 (68.0)	154 (8.0)	**<.001**
	Yes	723 (100.0)	108 (14.9)	487 (67.4)	128 (17.7)	
**Anxiety symptoms, n (%)**						
	No	1693 (100.0)	423 (25.0)	1130 (66.8)	140 (8.3)	**<.001**
	Yes	938 (100.0)	143 (15.3)	653 (69.6)	142 (15.1)	
Social status						
**Feelings of loneliness, n (%)**						
	No	1954 (100.0)	472 (24.2)	1311 (67.1)	171 (8.8)	**<.001**
	Yes	684 (100.0)	95 (13.9)	479 (70.0)	110 (16.1)	
**Usual social support, n (%)**						
	No	596 (100.0)	138 (23.2)	374 (62.8)	84 (14.1)	**.001**
	Yes	1811 (100.0)	378 (20.9)	1266 (69.9)	167 (9.2)	
**Emotional support, n (%)**						
	0	255 (100.0)	51 (20.0)	162 (63.5)	42 (16.5)	**.001**
	1 - 2	398 (100.0)	74 (18.6)	269 (67.6)	55 (13.8)	
	3	1974 (100.0)	440 (22.3)	1351 (68.4)	183 (9.3)	
**Previous participation in group activities, n (%)**						
	No	1112 (100.0)	263 (23.7)	716 (64.4)	133 (12.0)	**.004**
	Yes	1495 (100.0)	297 (19.9)	1054 (70.5)	144 (9.6)	
**Having a dog, n (%)**						
	No	2508 (100.0)	533 (21.3)	1697 (67.7)	278 (11.1)	**.007**
	Yes	130 (100.0)	36 (27.7)	90 (69.2)	4 (3.1)	
**Having a cat, n (%)**						
	No	2301 (100.0)	488 (21.2)	1577 (68.5)	236 (10.3)	.051
	Yes	337 (100.0)	81 (24.0)	210 (62.3)	46 (13.6)	
**Unfamiliarity with communication technology, n (%)**						
	No	1883 (100.0)	458 (24.3)	1252 (66.5)	173 (9.2)	**<.001**
	Yes	729 (100.0)	105 (14.4)	517 (70.9)	107 (14.7)	
**Death of a partner in the past year, n (%)**						
	No	2561 (100.0)	550 (21.5)	1735 (67.7)	276 (10.8)	.607
	Yes	58 (100.0)	12 (20.7)	42 (72.4)	4 (6.9)	

* Self-reported experience defined as "Positive", "Neutral", and "Negative" in participants reporting having experienced the lockdown as « not at all difficult », « somewhat difficult », and « very or extremely difficult », respectively.

†P-values from ANOVA test for age, and from Pearson's chi2-test or Fischer exact test when expected frequencies <5, for categorical variables.

‡BADL: basic activities of daily living (bathing, dressing, toileting, transferring, feeding and continence)

For peer review only

For peer review only - http://bmjopen.bmj.com/site/about/guidelines.xhtml

‖ IADL: instrumental activities of daily living (ability to use the telephone, to use transportations, prepare food, groceries, do the housekeeping, handle medications and manage finances).

Missing values (n): Education level (3), Financial difficulties (1), Living alone (8), Home type (6), Home location (22), Home characteristics (5), Comorbidities (13), BADL impairment (6), IADL impairment (36), Walking difficulty (18), Fear of falling (4), Memory difficulties (12), Depressive symptoms (3), Anxiety symptoms (11), Feelings of loneliness (4), Usual social support (235), Emotional support (15), Previous participation in group activities (35), Having a dog (4), Having a cat (4), Unfamiliarity with communication technology (30), Death of a partner in the past year (23).

Participants' experience of the lockdown did not differ according to their age (P=.434) and education level (P=.245). However, experience of the lockdown differed according to several other characteristics, some of which showed monotonically increasing or decreasing relationships, whereas others showed U- or inverted U-shaped associations.

Among **socio-demographic** characteristics, women were less likely than men to report a positive experience of the lockdown (18.8% vs 25.7%), and more likely to report a negative one (11.9% vs 8.8%, P<.001). By contrast, participants experiencing financial difficulties reported a negative experience more often than their peers did (13.2% vs 9.8%, P=.035).

Most characteristics reflecting a comfortable **living environment** (e.g., living in a house, having a terrace and/or garden) were also significantly associated with both higher rates of a positive experience and lower rates of a negative one (P <.001). By contrast, living alone was associated with higher proportions of both a negative and a positive experience than living with others (P=.001).

Similarly, most **health status** characteristics showed very important and significant inter-groups differences. Participants with poor self-rated health (P<.001), comorbidities (P=.001), BADL impairment (P=.013), IADL impairment (P<.001), fear of falling (P<.001), memory difficulties (P=.002), depressive symptoms (P<.001) and anxiety symptoms (P<.001) reported

For peer review only - http://bmjopen.bmj.com/site/about/guidelines.xhtml

more often a negative experience of the lockdown and less often a positive one than their counterparts.

Finally, **social status** characteristics also showed monotonic trends with a lower proportion of a positive experience and a higher proportion of a negative experience among participants feeling lonely (P<.001), without emotional support (P=.001), not having a dog (P=.007) and unfamiliar with communication technology (P<.001). The lack of social support (P=.001) and previous participation in group activities (P=.004) were associated with higher proportions of both negative and positive experiences and a lower proportion of a neutral one.

Table 2 shows the results of the **bivariate multinomial logistic regression**, comparing the groups reporting a positive experience and a negative experience to the neutral group (reference group).

Table 2. Results from bivariable multinomial logistic regression investigating participants' characteristics associated with a positive ("not at all difficult") and a negative ("very or extremely difficult") experience of the lockdown, using participants with a neutral ("slightly difficult") experience as reference group

	Experience of the lockdown					
	Positive (ref. neutral)			Negative (ref. neutral)		
	RRR	95% CI	P-value*	RRR	95% CI	P-value*
Socio-demographic						
**Age**	0.99	0.96 – 1.01	.223	1.00	0.97 – 1.03	.840
**Gender**						
Men	ref.			ref.		
Women	**0.69**	**0.57 – 0.84**	**<.001**	1.29	0.99 – 1.68	.064
**Education level**						
Basic compulsory	ref.			ref.		
Apprenticeship	0.86	0.65 – 1.15	.319	0.75	0.52 – 1.07	.107
Post-compulsory schooling	0.96	0.73 – 1.27	.784	**0.70**	**0.49 – 0.99**	**.045**
**Financial difficulties**						
No	ref.			ref.		
Yes	1.08	0.87 – 1.34	.474	**1.43**	**1.09 – 1.88**	**.010**
Living environment						
**Living alone**	ref.					
No	**1.34**	**1.11 – 1.62**		ref.	**1.09 – 1.81**	

For peer review only - http://bmjopen.bmj.com/site/about/guidelines.xhtml

Yes			.002	1.41		.008
**Home type**						
Apartment	ref.			ref.		
House	1.28	0.99 – 1.67	.062	**0.38**	**0.22 – 0.65**	**<.001**
**Home location**						
City	ref.			ref.		
Suburbs/rural areas	1.16	0.92– 1.45	.203	0.73	0.52 – 1.03	.070
**Home characteristics**						
No balcony, terrace or garden	0.98	0.64 – 1.52	.944	0.69	0.38 – 1.25	.222
A balcony	ref.			ref.		
A terrace and/or garden	1.17	0.96 – 1.43	.128	**0.48**	**0.35 – 0.66**	**<.001**
Health status						
**Self-rated health**						
Very good / good	ref.			ref.		
Average / poor / very poor	**0.68**	**0.55 – 0.84**	**<.001**	**1.87**	**1.45 – 2.41**	**<.001**
**Comorbidities**						
None	ref.			ref.		
1	**0.74**	**0.60 – 0.91**	**.005**	1.29	0.97 – 1.72	.079
2+	**0.66**	**0.50 – 0.86**	**.002**	1.30	0.92 – 1.83	.134
**BADL impairment**						
Independent	ref.			ref.		
Diff. / help for at least 1	**0.75**	**0.58 – 0.98**	**.034**	1.29	0.95 – 1.75	.104
**IADL impairment**						
Independent	ref.			ref.		
Diff. / help for at least 1	**0.59**	**0.48 – 0.71**	**<.001**	**1.77**	**1.36 – 2.32**	**<.001**
**Walking difficulty**						
No	ref.			ref.		
Yes	0.99	0.74 – 1.32	.946	1.21	0.85 – 1.73	.287
**Fear of falling**						
No	ref.			ref.		
Yes	**0.57**	**0.47 – 0.69**	**<.001**	**1.92**	**1.45 – 2.54**	**<.001**
**Homebound status**						
Non-homebound	ref.			ref.		
Semi-homebound	1.01	0.80 – 1.26	.953	0.92	0.67 – 1.25	.597
Homebound	2.22	0.84 – 5.87	.107	1.25	0.27 – 5.74	.775
**Memory difficulties**						
No	ref.			ref.		
Yes	0.82	0.63 – 1.09	.170	**1.58**	**1.16 – 2.15**	**.004**
**Depressive symptoms**						
No	ref.			ref.		
Yes	**0.63**	**0.50 – 0.79**	**<.001**	**2.22**	**1.72 – 2.87**	**<.001**
**Anxiety symptoms**						
No	ref.			ref.		
Yes	**0.59**	**0.47 – 0.72**	**<.001**	**1.76**	**1.36 – 2.26**	**<.001**
Social status						
**Feelings of loneliness**						
No	ref.			ref.		
Yes	**0.55**	**0.43 – 0.70**	**<.001**	**1.76**	**1.36 – 2.29**	**<.001**

For peer review only - http://bmjopen.bmj.com/site/about/guidelines.xhtml

**Usual social support**							
	No	ref.			ref.		
	Yes	0.81	0.65 – 1.01	.067	**0.59**	**0.44 – 0.78**	**<.001**
**Emotional support**							
	0	ref.			ref.		
	1-2	0.87	0.58 – 1.31	.515	0.79	0.50 – 1.23	.297
	3	1.03	0.74 – 1.44	.841	**0.52**	**0.36 – 0.76**	**<.001**
**Previous participation in group activities**							
	No	ref.			ref.		
	Yes	**0.77**	**0.63 – 0.93**	**.007**	**0.74**	**0.57 – 0.95**	**.018**
**Having a dog**							
	No	ref.			ref.		
	Yes	1.27	0.85 – 1.90	.234	**0.27**	**0.10 - 0.74**	**.011**
**Having a cat**							
	No	ref.			ref.		
	Yes	1.25	0.95 – 1.64	.117	**1.46**	**1.03 – 2.07**	**.031**
**Unfamiliarity with communication technology**							
	No	ref.			ref.		
	Yes	**0.56**	**0.44 – 0.70**	**<.001**	**1.50**	**1.15 – 1.95**	**.003**
**Death of a partner in the past year**							
	No	ref.			ref.		
	Yes	0.90	0.47 – 1.72	.754	0.60	0.21 – 1.68	.331

Notes: RRR relative risk ratio; CI confidence interval.
* P-values from multinomial logistic regression.

The relative risk of reporting a **positive experience of the lockdown** – rather than a neutral one – was 34% **higher** among participants living alone (RRR 1.34, 95%CI 1.11–1.62), but 31% **lower** among women (RRR 0.69, 95%CI 0.57–0.84). It was also lower in participants with average, poor or very poor self-rated health, comorbidities, impairment in BADL and IADL, fear of falling, depressive symptoms, anxiety symptoms, a feeling of loneliness, previous participation in group activities, or who were unfamiliar with communication technology (P<.05 for all associations).

The relative risk of reporting a **negative experience of the lockdown** – rather than a neutral one – **increased** among participants who reported financial difficulties (RRR 1.43, 95%CI 1.09–1.88), but also in participants living alone, who rated their health as average, poor or very

For peer review only - http://bmjopen.bmj.com/site/about/guidelines.xhtml

For peer review only

poor, reported IADL impairment, fear of falling, memory difficulties, depressive symptoms, anxiety symptoms, feelings of loneliness, having a cat, and being unfamiliar with communication technology (P<.05 for all associations). On the contrary, the relative risk **decreased** among participants with a post-compulsory education level (RRR 0.70, 95%CI 0.49–0.99), but also in participants who lived in a house, had access to a terrace and/or garden, benefited from usual social support, reported at least 3 sources of emotional support, had previously participated in group activities and owned a dog (P<.05 for all associations).

Interestingly, some characteristics showed significant associations in a consistent direction, with increased relative risks of a negative experience, on the one hand, and decreased relative risks of a positive experience on the other hand. For instance, participants who reported fear of falling had a 43% lower relative risk of reporting a positive experience (RRR 0.57, 95%CI 0.47–0.69) and were almost twice more likely to report a negative experience (RRR 1.92, 95%CI 1.45–2.54) of the lockdown as those without any fear of falling. Similar associations with both significant positive and negative experience were also observed for self-rated health, IADL impairment, depressive symptoms, anxiety symptoms, feelings of loneliness, and being unfamiliar with communication technology.

Two characteristics showed a U-shaped association with the experience of the lockdown. Participants living alone had a higher relative risk to report both a positive (RRR 1.34, 95%CI 1.11–1.62) and a negative (RRR 1.41, 95%CI 1.09–1.81) experience rather than a neutral one. Inversely, previous participation in group activities (reported by 59.5% of the total population) was associated with decreased relative risks of reporting a positive (RRR 0.77, 95%CI 0.63–0.93) or a negative (RRR 0.74, 95%CI 0.57–0.95) experience of the lockdown.

Figure 2 shows the results of the **multivariable multinomial logistic regression** (for more details, see Supplementary – Table 3). The relative risk of reporting a **positive experience** –

For peer review only - http://bmjopen.bmj.com/site/about/guidelines.xhtml

rather than a neutral one – remained **higher** for participants who lived alone (RRR 1.93, 95%CI 1.52–2.46) or in a house (RRR 1.49, 95%CI 1.03–2.16), and **lower** for women (RRR 0.75, 95%CI 0.59–0.95), for participants who reported IADL impairment (RRR 0.78, 95%CI 0.61–0.99), fear of falling (RRR 0.68, 95%CI 0.54–0.86), feelings of loneliness (RRR 0.67, 95%CI 0.49–0.91), for those who received usual social support (RRR 0.71, 95%CI 0.54–0.93), and for those who had previously participated in group activities (RRR 0.74, 95%CI 0.59–0.92), or were unfamiliar with communication technology (RRR 0.69, 95%CI 0.52–0.91).

Few characteristics remained independently associated with a **negative experience** of the lockdown. The relative risk of the latter – compared to a neutral one – **increased** among participants with fear of falling (RRR 1.52, 95%CI 1.07–2.15), and **decreased** among those who had access to a terrace and/or garden (RRR 0.66, 95%CI 0.44–0.99), or owned a dog (RRR 0.32, 95%CI 0.11–0.9).

In the multivariable model, fear of falling was the only factor that remained significantly associated in **a consistent direction**, with an increased relative risk of a negative experience (RRR 1.52, 95%CI 1.07–2.15) and a decreased relative risk of a positive experience (RRR 0.68, 95%CI 0.54–0.86) compared to the neutral group.

None of the tested interactions were significant. The sensitivity analysis conducted using the multivariate imputation approach produced very similar results (see Supplementary – Table 3bis).

For peer review only

DISCUSSION

This study examined the experience of the COVID-19 partial lockdown in a community-dwelling Swiss older population. Its unique contribution was to highlight which participants' characteristics were independently associated with a positive and a negative experience, respectively. Results confirmed our initial hypothesis that most participants (i.e., about two thirds) would report a neutral experience of the lockdown and describe this period as "somewhat difficult". By contrast, only about one in ten older people reported a negative experience of the lockdown, describing it as "very" or "extremely" difficult. At the other end of the spectrum, more than a fifth of the participants reported a positive experience and described the lockdown as "not difficult at all". Most of the observed associations were in line with our hypotheses: participants who lived in a more spacious environment (living in a house) and/or with outdoor access (having a terrace and/or garden) were significantly less likely than their counterparts to report a negative rather than a neutral experience. Reversely, participants with health problems (fear of falling and functional difficulties) as well as those who were socially vulnerable (who reported feelings of loneliness, receiving usual social support and being unfamiliar with communication technology) were significantly less likely to report a positive experience.

Several notable associations were rather unexpected. For instance, contrary to our initial hypothesis, living alone turned out to be associated with a higher relative risk of a positive experience. This result contrasts with another study that identified living alone as a vulnerability factor during a lockdown (26). The possibility to adjust for the feeling of loneliness might explain the current study's finding in that regard. Indeed, participants who expressed feelings of loneliness were significantly less likely than their counterparts to report a positive rather than

For peer review only - http://bmjopen.bmj.com/site/about/guidelines.xhtml

a neutral experience – a finding that is consistent with the results of another Swiss study (14). This hypothesis is further supported by the observation that participants who had usual social support and had previously participated in group activities were more likely to report a negative experience of the lockdown. Overall, these results suggest that older participants who were socially more active and involved, and those who already felt lonely were less able to cope with the lockdown than those who were already living alone before the pandemic. Indeed, older people who still live alone in their home tend to be more mobile, more independent and autonomous, and may not be particularly prone to social isolation (27). However, this hypothesis is not supported in this study. Indeed 36.3% of participants who lived alone reported feelings of loneliness, compared to 17.6% of those who lived together with other people. In this regard, the observed relationship between participants' unfamiliarity with communication technology and a reduced chance of reporting a positive experience of the lockdown is particularly interesting. Keeping in touch with their social network during the lockdown was crucial for older people, as some reported feeling cut off from society (28). Communication technology was identified as a coping strategy in hospitals and nursing homes. Health teams often used the technology of tablets to help older people keep in touch with their families in times when visits were forbidden. Results from the current study extend current knowledge as findings about the role of information technology and Internet access in preventing older adults from feeling lonely and socially disconnected are discordant (29–31). Our observation provides further evidence of the need to foster the training and supervision of older people in using communication technology to reduce their loneliness and social isolation.

The lack of association between participants' age and their experience of the lockdown was also contrary to our hypothesis. As some studies suggest, the relationship between age and coping mechanisms might be more complex, as older people tend to show more resilience than younger ones during stressful times (12). Similarly, the lack of association between a partner's

For peer review only

death in the past year and the experience of the lockdown might be surprising. However, such a lack likely resulted from the low (2.2%) incidence of widowhood in the study population. Unfortunately, the lack of data on the specific timing and cause of death made it impossible to investigate the likelihood of an association in a subgroup whose spouse died recently, and/or from a SARS-CoV-2 infection.

Interestingly, this study further contributed to highlighting the independent association between the experience of the lockdown and several characteristics of the participants' living environment. Although none of these characteristics showed a significant independent association in a consistent direction (i.e., a decreased risk of a negative experience and an increased chance of a positive experience or reversely), living in a house and having access to a terrace and/or garden appeared to influence the experience of the lockdown in one of the hypothesised directions. Fortunately, few participants (5.3%) had no access to a balcony, a terrace and/or garden – a factor worth examining in case of another lockdown in the future.

The robust observation of the association between the participants' fear of falling and their experience of the lockdown is meaningful, too. Indeed, fear of falling was the only variable that remained independently associated in a consistent direction: fearful participants were both at an increased relative risk of reporting a negative experience and at a lower relative risk of reporting a positive experience. Notably, this association remained, even after extensive adjustment for other measurements of the health status (including anxiety), as well as for socio-demographic, living environment and social status characteristics. The reason for this may be that the fear of falling reflects a state of increased combined mental and physical vulnerability, as also suggested by other studies that found a robust association with outdoor mobility and quality of life in community-dwelling older people (32–34). Further research should investigate whether the fear of falling should be addressed through interventions, such as exercise, primary fall prevention, as well as behavioral and cognitive therapies (35).

For peer review only - http://bmjopen.bmj.com/site/about/guidelines.xhtml

Finally, the observation that dog owners were particularly unlikely to report a negative experience, whereas cat owners were likely to do so, deserves a comment. This suggests that the relationship between dog ownership and the experience of the lockdown occurred through enhanced mobility rather than merely through possessing a pet animal. This goes in line with a recent Japanese study (36), which showed an association between dog ownership and a reduced risk of disability, possibly because of the resulting moderate physical activity maintained. Alternative explanations could also be provided by previously reported differences between cat and dog owners in feelings of loneliness and social isolation, as well as by differences in personality patterns (37,38). To encourage older people to practise dog walking could be an interesting option in times of a lockdown – and probably also in general – as it might prevent them from developing disability and frailty, but also help them enjoy more social contact and reduce feelings of loneliness.

Strengths and limitations

A major strength of this population-based study is the use of postal – rather than online – questionnaires, for it allowed to include the subgroup of individuals who are uncomfortable with the use of communication technology. The fact that participants completed the questionnaires during the lockdown excludes any memory bias. Furthermore, the design of the study allowed to link the data collected during the lockdown with key characteristics routinely assessed in the Lc65+ study.

This study has certain limitations. First, because the results were obtained from community-dwelling adults aged 71 to 86 years, they cannot be generalised to all older individuals. Second, the most vulnerable participants had to be excluded from the study due to missing data or institutionalisation. Fortunately, excluded individuals represented only a very low percentage

For peer review only - http://bmjopen.bmj.com/site/about/guidelines.xhtml

of the total sample (7.5%). In terms of external validity, this study focused on a partial lockdown. During the same period, many other countries implemented a stricter or even a total lockdown. Furthermore, this study focused on the specific part of the older population that lives in the community, is in relatively good health, and is relatively independent. Accordingly, it might be that the partial lockdown was less restrictive for this category of older people regarding social interactions than for older people who live in a nursing home or who visit day-care facilities. Finally, data on socio-demographic, living environment, health, and social characteristics stem mainly from the 2019 questionnaire. Although living environment and socio-demographic characteristics do not appear particularly prone to substantial variations from one year to another, we cannot formally exclude minor changes in health and social characteristics from 2019 to 2020. Nevertheless, if preventive interventions are to be implemented in the future to limit the impact of stringent distancing measures, data available to target those interventions will have been collected before the introduction of the measures for a timely and efficient approach.

Future implications

This study identified that many participants' characteristics regarding socio-demography, living environment, health and social status were related with their experience of the lockdown. From a public health perspective, this study further highlights the importance of targeted interventions in reducing the impact of another potential lockdown in the future. For example, maintaining a higher level of mobility could be achieved by actions such as accompanying older people for outside walks or more specifically by means of actions that alleviates the fear of falling (e.g. exercise, behavioral and cognitive therapy) even if such interventions must stay in line with sanitary measures and individuals' security. Modifying the physical environment and

For peer review only - http://bmjopen.bmj.com/site/about/guidelines.xhtml

promoting activities such as walking a dog could also be recommended to address social isolation. Finally, this study's findings add to previous evidence of the necessary empowerment of older people, especially of those who express feelings of loneliness; by learning how to master and use communication technology, benefit from more social and emotional support, and keep in touch with their relatives.

For peer review only

For peer review only - http://bmjopen.bmj.com/site/about/guidelines.xhtml

Author contributions DMG drafted the manuscript, SF conducted the analyses, YH supervised the work, designed and conducted the data collection as the principal investigator of the Lc65+ study, CB and LSB designed and supervised the work. All authors (DMG, SF, YH, CB, LSB, JS) read, revised and approved the final manuscript.

Acknowledgements We sincerely thank all participants of the Lc65+ study. We are also grateful to the Lc65+ collaborators for their involvement in the data collection, as well as to Prof. Brigitte Santos-Eggimann for her contribution to the conception of the COVID-19 specific questionnaire.

Funding For this research, the authors have not declared any grant from any funding agency in the public, commercial or not-for-profit sectors.

From the beginning, the Lc65+ study has been financed exclusively by public funds or not-for-profit organisations. It is currently funded by the Public Health Department of canton Vaud, the Centre for Primary Care and Public Health (Unisanté) and by research grants from the Loterie Romande (not-for-profit organisation supporting research and social projects) and the Esther Locher-Gurtner Foundation.

Conflicts of interest None declared

Ethics approval The Cantonal Human Research Ethical Committee approved the Lausanne Cohort Lc65+ initial study protocol (Protocol (19/04), decision letter: 23 February 2004), the successive amendments and the COVID-19 questionnaire for follow-up. All participants gave their written informed consent.

Data availability statement All data relevant to the study are included in the article or uploaded as supplementary information.

For peer review only - http://bmjopen.bmj.com/site/about/guidelines.xhtml

REFERENCES

1. Pfefferbaum B, North CS. Mental Health and the Covid-19 Pandemic. N Engl J Med [Internet]. 2020 Apr 13 [cited 2021 Feb 5]; Available from: https://www.nejm.org/doi/10.1056/NEJMp2008017

2. Tull MT, Edmonds KA, Scamaldo KM, Richmond JR, Rose JP, Gratz KL. Psychological Outcomes Associated with Stay-at-Home Orders and the Perceived Impact of COVID-19 on Daily Life. Psychiatry Res. 2020 Jul;289:113098.

3. Qiu J, Shen B, Zhao M, Wang Z, Xie B, Xu Y. A nationwide survey of psychological distress among Chinese people in the COVID-19 epidemic: implications and policy recommendations. Gen Psychiatry [Internet]. 2020 Mar 6 [cited 2021 Feb 5];33(2). Available from: https://www.ncbi.nlm.nih.gov/pmc/articles/PMC7061893/

4. Gandre C., Coldefy M., Rochereau T. / collab. Les inégalités face au risque de détresse psychologique pendant le confinement : Premiers résultats de l'enquête COCLICO du 3 au 14 avril 2020. Quest Econ SANTE IRDES. 2020 Jun;(249):8p.

5. Wang C, Pan R, Wan X, Tan Y, Xu L, Ho CS, et al. Immediate Psychological Responses and Associated Factors during the Initial Stage of the 2019 Coronavirus Disease (COVID-19) Epidemic among the General Population in China. Int J Environ Res Public Health [Internet]. 2020 Mar [cited 2021 Feb 5];17(5). Available from: https://www.ncbi.nlm.nih.gov/pmc/articles/PMC7084952/

6. Iob E, Frank P, Steptoe A, Fancourt D. Levels of Severity of Depressive Symptoms Among At-Risk Groups in the UK During the COVID-19 Pandemic. JAMA Netw Open. 2020 Oct 1;3(10):e2026064.

7. Girdhar R, Srivastava V, Sethi S. Managing mental health issues among elderly during COVID-19 pandemic. 2020;4.

8. Pierce M, Hope H, Ford T, Hatch S, Hotopf M, John A, et al. Mental health before and during the COVID-19 pandemic: a longitudinal probability sample survey of the UK population. Lancet Psychiatry. 2020 Oct;7(10):883–92.

9. González-Sanguino C, Ausín B, Castellanos MÁ, Saiz J, López-Gómez A, Ugidos C, et al. Mental health consequences during the initial stage of the 2020 Coronavirus pandemic (COVID-19) in Spain. Brain Behav Immun. 2020 Jul;87:172–6.

10. Luo M, Guo L, Yu M, Jiang W, Wang H. The psychological and mental impact of coronavirus disease 2019 (COVID-19) on medical staff and general public - A systematic review and meta-analysis. Psychiatry Res. 2020 Sep;291:113190.

11. Benke C, Autenrieth LK, Asselmann E, Pané-Farré CA. Lockdown, quarantine measures, and social distancing: Associations with depression, anxiety and distress at the beginning of the COVID-19 pandemic among adults from Germany. Psychiatry Res. 2020 Nov;293:113462.

For peer review only - http://bmjopen.bmj.com/site/about/guidelines.xhtml

12. López J, Perez-Rojo G, Noriega C, Carretero I, Velasco C, Martinez-Huertas JA, et al. Psychological well-being among older adults during the COVID-19 outbreak: a comparative study of the young–old and the old–old adults. Int Psychogeriatr. 2020 May 17;1–6.

13. Röhr S, Reininghaus U, Riedel-Heller SG. Mental wellbeing in the German old age population largely unaltered during COVID-19 lockdown: results of a representative survey. BMC Geriatr. 2020 Nov 23;20(1):489.

14. Macdonald B, Hülür G. Well-Being and Loneliness in Swiss Older Adults During the COVID-19 Pandemic: The Role of Social Relationships. The Gerontologist. 2021 Mar 1;61(2):240–50.

15. Falvo I, Zufferey MC, Albanese E, Fadda M. Lived experiences of older adults during the first COVID-19 lockdown: A qualitative study. PLOS ONE. 2021 Jun 23;16(6):e0252101.

16. Henchoz Y, Blanco JM, Fustinoni S, Nanchen D, Büla C, Seematter-Bagnoud L, et al. Cohort Profile: The Lausanne cohort 65+ (Lc65+). Int J Epidemiol. 2021 Nov 29;dyab245.

17. Santos-Eggimann B, Karmaniola A, Seematter-Bagnoud L, Spagnoli J, Büla C, Cornuz J, et al. The Lausanne cohort Lc65+: a population-based prospective study of the manifestations, determinants and outcomes of frailty. BMC Geriatr. 2008 Aug 18;8:20.

18. Katz S, Ford AB, Moskowitz RW, Jackson BA, Jaffe MW. Studies of Illness in the Aged: The Index of ADL: A Standardized Measure of Biological and Psychosocial Function. JAMA. 1963 Sep 21;185(12):914–9.

19. Lawton MP, Brody EM. Assessment of Older People: Self-Maintaining and Instrumental Activities of Daily Living1. The Gerontologist. 1969 Oct 1;9(3_Part_1):179–86.

20. MacKay S, Ebert P, Harbidge C, Hogan DB. Fear of Falling in Older Adults: A Scoping Review of Recent Literature. Can Geriatr J. 2021 Dec 1;24(4):379–94.

21. Whooley MA. Screening for Depression—A Tale of Two Questions. JAMA Intern Med. 2016 Apr 1;176(4):436.

22. Boyer P, Bisserbe JC, Weiller E. How efficient is a screener? A comparison of the PRIME-MD patient questionnaire with the SDDS-PC screen. Int J Methods Psychiatr Res. 1998;7(1):27–32.

23. Lubben J, Blozik E, Gillmann G, Iliffe S, von Renteln Kruse W, Beck JC, et al. Performance of an Abbreviated Version of the Lubben Social Network Scale Among Three European Community-Dwelling Older Adult Populations. The Gerontologist. 2006 Aug;46(4):503–13.

24. Sherbourne CD, Stewart AL. The MOS social support survey. Soc Sci Med 1982. 1991;32(6):705–14.

For peer review only

25. Rubin DB. Multiple Imputation for Nonresponse in Surveys. In: Multiple Imputation for Nonresponse in Surveys [Internet]. John Wiley & Sons, Ltd; 1987 [cited 2021 Nov 16]. Available from: https://onlinelibrary.wiley.com/doi/abs/10.1002/9780470316696.fmatter

26. Zaninotto P, Iob E, Demakakos P, Steptoe A. Immediate and Longer-Term Changes in the Mental Health and Well-being of Older Adults in England During the COVID-19 Pandemic. JAMA Psychiatry. 2022 Feb;79(2):1–9.

27. Michael YL, Berkman LF, Colditz GA, Kawachi I. Living Arrangements, Social Integration, and Change in Functional Health Status. Am J Epidemiol. 2001 Jan 15;153(2):123–31.

28. Portacolone E, Chodos A, Halpern J, Covinsky KE, Keiser S, Fung J, et al. The Effects of the COVID-19 Pandemic on the Lived Experience of Diverse Older Adults Living Alone With Cognitive Impairment. Bowers BJ, editor. The Gerontologist. 2021 Feb 23;61(2):251–61.

29. Eghtesadi M. Breaking Social Isolation Amidst COVID-19: A Viewpoint on Improving Access to Technology in Long-Term Care Facilities. J Am Geriatr Soc. 2020 May;68(5):949–50.

30. Noone C, McSharry J, Smalle M, Burns A, Dwan K, Devane D, et al. Video calls for reducing social isolation and loneliness in older people: a rapid review. Cochrane Database Syst Rev. 2020 May 22;2020(5):CD013632.

31. Bonsaksen T, Thygesen H, Leung J, Ruffolo M, Schoultz M, Price D, et al. Video-Based Communication and Its Association with Loneliness, Mental Health and Quality of Life among Older People during the COVID-19 Outbreak. Int J Environ Res Public Health. 2021 Jun 10;18(12):6284.

32. Schoene D, Heller C, Aung YN, Sieber CC, Kemmler W, Freiberger E. A systematic review on the influence of fear of falling on quality of life in older people: is there a role for falls? Clin Interv Aging. 2019 Apr;Volume 14:701–19.

33. Scheffer AC, Schuurmans MJ, van Dijk N, van der Hooft T, de Rooij SE. Fear of falling: measurement strategy, prevalence, risk factors and consequences among older persons. Age Ageing. 2008 Jan;37(1):19–24.

34. Spaltenstein J, Bula C, Santos-Eggimann B, Krief H, Seematter-Bagnoud L. Factors associated with going outdoors frequently: a cross-sectional study among Swiss community-dwelling older adults. BMJ Open [Internet]. 2020 Aug 24 [cited 2021 Jan 15];10(8). Available from: https://www.ncbi.nlm.nih.gov/pmc/articles/PMC7449269/

35. Kendrick D, Kumar A, Carpenter H, Zijlstra GAR, Skelton DA, Cook JR, et al. Exercise for reducing fear of falling in older people living in the community. Cochrane Database Syst Rev. 2014 Nov 28;2014(11):CD009848.

36. Taniguchi Y, Seino S, Headey B, Hata T, Ikeuchi T, Abe T, et al. Evidence that dog ownership protects against the onset of disability in an older community-dwelling Japanese population. Mogi M, editor. PLOS ONE. 2022 Feb 23;17(2):e0263791.

For peer review only - http://bmjopen.bmj.com/site/about/guidelines.xhtml

37. Hajek A, König HH. How do cat owners, dog owners and individuals without pets differ in terms of psychosocial outcomes among individuals in old age without a partner? Aging Ment Health. 2020 Oct 2;24(10):1613–9.

38. Schulz C, König HH, Hajek A. Differences in Self-Esteem Between Cat Owners, Dog Owners, and Individuals Without Pets. Front Vet Sci. 2020 Sep 2;7:552.

For peer review only

For peer review only - http://bmjopen.bmj.com/site/about/guidelines.xhtml

FIGURES' CAPTIONS

Figure 1. Flow chart describing the selection of the study population.

Figure 2. Results from the multivariable multinomial logistic regression analysis investigating participants' characteristics associated with a self-reported positive (green boxes, "not difficult at all") and negative (red triangles, "very or extremely difficult") experience of the lockdown, using participants with neutral ("slightly difficult") experience as a reference group

Relative risk ratio (RRR) from the multivariable multinomial logistic regression, CI: confidence interval

*** P<.001, ** P<.01, * P<.05

For peer review only - http://bmjopen.bmj.com/site/about/guidelines.xhtml

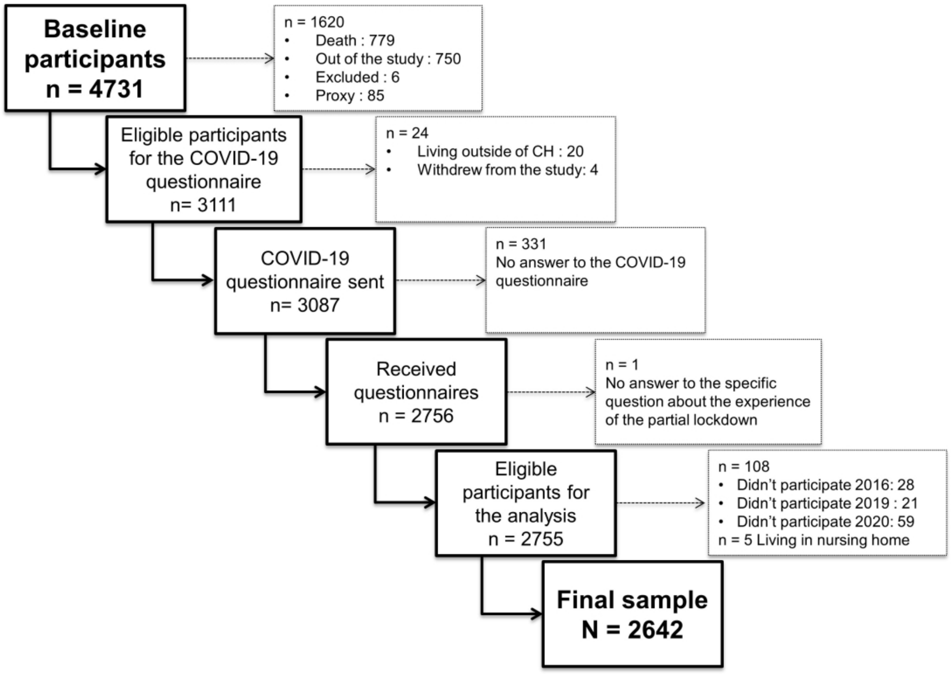

254x190mm (300 x 300 DPI)

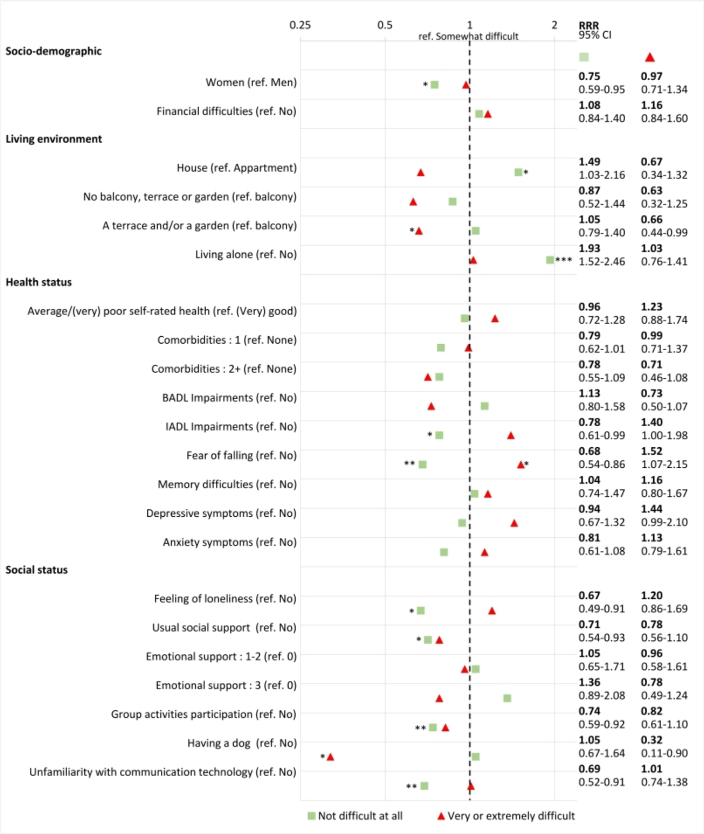

209x297mm (300 x 300 DPI)

Supplementary material

Supplementary Table 1. Source of study variables in the questionnaires and time of measurement

1. Katz S, Ford AB, Moskowitz RW, Jackson BA, Jaffe MW. Studies of Illness in the Aged: The Index of ADL: A Standardized Measure of Biological and Psychosocial Function. JAMA. 1963 Sep 21;185(12):914–9.

2. Lawton MP, Brody EM. Assessment of Older People: Self-Maintaining and Instrumental Activities of Daily Living1. The Gerontologist. 1969 Oct 1;9(3_Part_1):179–86.

3. MacKay S, Ebert P, Harbidge C, Hogan DB. Fear of Falling in Older Adults: A Scoping Review of Recent Literature. Can Geriatr J. 2021 Dec 1;24(4):379–94.

4. Whooley MA. Screening for Depression—A Tale of Two Questions. JAMA Intern Med. 2016 Apr 1;176(4):436.

5. Boyer P, Bisserbe JC, Weiller E. How efficient is a screener? A comparison of the PRIME-MD patient questionnaire with the SDDS-PC screen. Int J Methods Psychiatr Res. 1998;7(1):27–32.

6. Sherbourne CD, Stewart AL. The MOS social support survey. Soc Sci Med 1982. 1991;32(6):705–14.

Abbreviation	Questionnaire	Time period of questionnaire completion
qlock	Lockdown questionnaire	17.04.2020 – 31.05.2020
q2016	Yearly questionnaire 2016	01.01.2016 – 31.12.2016
q2019	Yearly questionnaire 2019	01.01.2019 – 31.12.2019
q2020	Yearly questionnaire 2020	01.01.2020 – 31.12.2020
qbase	Baseline questionnaire	Time of enrollment for each cohort i.e.:<br>– Cohort 1: 01.01.2004 – 31.12.2004<br>– Cohort 2: 01.01.2009 – 31.12.2009<br>– Cohort 3: 01.01.2014 – 31.12.2014

**Outcome**	**Measure – Question in the lockdown questionnaire (qlock) April – May 2020**
**Experience of the lockdown**	qlock Q19 "To what extent is the lockdown currently difficult to cope with?"<br><br>With the choice of answers:<br><br>1. Not at all difficult<br>2. Slightly difficult<br>3. Very difficult or 4. Extremely difficult (grouped as one category)

**Characteristics**	**Measure – Questions in the yearly questionnaires (q2019, q2020, q2016), the baseline questionnaire (qbase) and lockdown questionnaire (qlock)**
**Age**	Mean (standard deviation)<br>2020 – birth year
**Gender**	Men / Women
**Education level**	qbase Q13 “What is the highest level of education you have completed?”<br><br>1. Compulsory schooling<br>2. Apprenticeship<br>3. Baccalaureate, high school diploma<br>4. Federal master's degree, professional diploma<br>5. University, college

For peer review only - http://bmjopen.bmj.com/site/about/guidelines.xhtml

	3 groups: 1 Basic compulsory/2 Apprenticeship/3 Post-compulsory schooling
**Frequency of going out of home (homebound status)**	q2019 Q31 (Q30 C2) "How frequently do you go out of your home?"<br>1. 5-7 days per week (non-homebound)<br>2. 3-4 days per week + 3. 1-2 days per week (grouped as one category: semi-homebound)<br>4. I almost never go out of my home (homebound)
**Living situation in 2020:**<br>**1. Living alone**<br>**2. Home type**<br>**3. Home location**<br>**4. Home characteristics**<br>**5. Having a pet animal**	1. qlock Q7 "What are your living arrangements during this lockdown period due to Covid?"<br>2 groups: answer 1 (alone) versus answer 2 (with … other persons)<br>2. qlock Q8a. "What are the characteristics of your living accommodation?"<br>apartment versus house<br>3. qlock Q8b. "What are the characteristics of your living accommodation?"<br>2 groups: in town; suburbs or country<br>4. qlock Q8c.+d. "What are the characteristics of your living accommodation?" balcony (yes/no) + terrace and/or garden (yes/no)<br>3 groups: no balcony/terrace/garden; having a balcony; having a terrace or a garden<br>5. q2020 Q61 (C2 56) "Do you live with a pet animal?" (yes/no)<br>q2020 Q62 (C2 57) "What animal(s) do you live with?"<br>a) cat(s)<br>b) dog(s)<br>c) other(s): specify<br>2 variables: Having a cat (yes/no) / Having a dog (yes/no)
**Indicators of low economic status (financial difficulties)**	1. q2019 Q51 (C2 Q45) "Do you currently receive state subsidies for your health insurance costs?" (yes/no)<br>2. q2019 Q52 (C2 Q46) "Do you currently receive complementary subsidies (PC) from the old age and survivor's insurance (AVS)?" (yes/no)<br>2 groups: absence of difficulties versus answering yes at one of the 2 questions
**Self-judgement of health status**	q2019, Q1 "In your opinion, what is your current state of health?"<br>answer 1+2 (very good and good) vs. 3+4+5 (average, poor, very poor)
**Comorbidities**	q2019 Q4: "During the past 12 months, were you treated, or did you suffer from any of the following health problems, diagnosed by a physician?"<br>-No illness (item 20) versus<br>-One of:<br>-Two or more of:<br>-Cardiac comorbidities: coronaropathy (3) + cardiac insufficiency (4) + valvular or cardiac muscle deficiency (5)<br>-TIA, stroke (6)<br>-Diabetes (7)<br>-Chronic pulmonary conditions (8) and asthma (9)

For peer review only

	-Osteoarticular conditions: osteoporosis (10) + fractures (11) + arthrosis or arthritis (12)<br>-Oncologic illness (13)<br>-Parkinson and Alzheimer (16) (17)
**Basic activities of daily living (BADL) and instrumental activities of daily living (IADL)**	q2019 Q43 (C2 Q40) "Do you have difficulties or receive help for the following activities due to physical, psychological, emotional or memory problems?" (bathing, getting dressed, toileting, transferring, feeding, continence (BADL); ability to use the telephone, to use transportations, prepare food, shop for groceries, do the housekeeping, prepare and take your medications, manage finances (IADL))<br><br>2 groups: independent BADL versus difficulties with at least 1 BADL (1)<br>2 groups: independent IADL versus difficulties with at least 1 IADL (2)
**Indicators of mobility:**<br>**1. Walking difficulties**<br>**2. Fear of falling**	1. q2019 Q35a (C2 Q34a) "Do you find it difficult to walk 100 meters for health reasons?" (yes/no)<br>2. q2019 Q37 (C2 Q36) "Do you fear to fall?" (yes/no) (3)
**Memory difficulties**	q2019 Q5 item 7, 8, 9 "Have you been disrupted since at least 6 months by memory gaps that affect your daily life or by concentration difficulties or decision-making difficulties?" (yes/no)
**Depressive symptoms (self-reported)**	1. q2019 Q17 "During the past 4 weeks, did you often feel sad, depressed or discouraged?" (yes/no)<br>2. q2019 Q18 "During the past 4 weeks, did you feel a loss of interest or pleasure in your usual activities?" (yes/no)<br><br>2 groups using Whooley's two questions (4): no symptoms versus presence of symptoms (sadness or apathy)
**Anxiety symptoms (self-reported)**	1. q2019 Q19 "During the past 4 weeks, did you often feel preoccupied and anxious?" (yes/no)<br>2. q2019 Q20 "During the past 4 weeks, how often did you feel calm, balanced, serene?" (rarely, never vs. other responses)<br><br>2 groups: no symptoms versus yes to question Q19 and/or sometimes/rarely/never to question Q20 adapted from the PRIME-MD screen (5)
**Feeling of loneliness**	q2019 Q21 "During the past 4 weeks, how often did you feel isolated?" (always, very often, often, sometimes, rarely, never)<br><br>2 groups: always/very often/often/sometimes versus rarely/never
**Usual social support**	1. q2016 Q44 (C2 Q32) With how many members of your family (partner, children, etc.) do you:<br>a) get in touch (visually or on the phone) at least once a month?<br>b) feel close enough to ask them for help when needed?<br>c) feel at ease to discuss private matters?<br><br>2. q2016 Q45 (C2 Q33) With how many friends or neighbors do you :<br>a) get in touch (visually or on the phone) at least once a month?<br>b) feel close enough to ask them for help when needed?<br>c) feel at ease to discuss private matters?<br><br>2 groups according to clinical cut point of Lubben Social Network Scale (2):<br>Socially supported (LSNS-6≥12) / Not socially supported (LSNS-6<12)

For peer review only

**Emotional Support**	1. q2016 Q46 (C2 Q34) How often can you count on someone to show you love or affection when needed? (always, very often, often, sometimes, rarely, never)<br>2. q2016 Q47 (C2 Q35) How often can you count on someone with whom you can share your intimate preoccupation when needed? (always, very often, often, sometimes, rarely, never)<br>3. q2016 Q48 (C2 Q36) How often can you rely on someone you love and for whom you are important? (always, very often, often, sometimes, rarely, never)<br><br>3 groups according to three items from the MOS social support survey (6): no emotional support declared as always, very often, often; 1-2 emotional support declared as always, very often, often; 3 emotional support declared as always, very often, often
**Familiarity with communication technology**	q2020 Q54 (C1 Q59) "Do you forgo certain activities because you are unfamiliar with technology?" (yes/no)
**Previous participation in group activities**	q2016 Q49 (C2 Q37) How many times a month do you participate in group activities (such as recreation activities, local activities, charitable associations, political or religious groups)<br><br>2 groups: 0 times versus > 0 times
**Death of a partner in the past year**	q2019 Q56 (C2 Q47) j: "During the past 12 months, did you have to face with the death of your spouse or partner?" (yes/no)

For peer review only - http://bmjopen.bmj.com/site/about/guidelines.xhtml

Supplementary Table 2. Comparisons of characteristics in participants included vs not included in the study

*P-values from Student's t-test for age and from Pearson's chi2 test for categorical variables.

	Total N=2755	Included N=2642	Not included N=113	P-value*
**Age, mean (sd)**	78.1 (4.2)	78.0 (4.2)	79.3 (4.6)	**.002**
**Gender, n (%)**				
Men	1093 (39.7)	1059 (40.1)	34 (30.1)	**.033**
Women	1662 (60.3)	1583 (59.9)	79 (69.9)	
**Education level, n (%)**				
Basic compulsory	438 (15.9)	404 (15.3)	34 (30.1)	**<.001**
Apprenticeship	1072 (39.0)	1032 (39.1)	40 (35.4)	
Post-compulsory schooling	1242 (45.1)	1203 (45.6)	39 (34.5)	
**Depressive symptoms, n (%)**				
No	1962 (72.0)	1916 (72.6)	46 (53.5)	**<.001**
Yes	763 (28.0)	723 (27.4)	40 (46.5)	
**Self-rated health, n (%)**				
Very good / good	1775 (65.1)	1734 (65.6)	41 (47.7)	**.001**
Average / poor / very poor	953 (34.9)	908 (34.4)	45 (52.3)	
**Living alone, n (%)**				
No	1527 (55.6)	1465 (55.6)	62 (55.4)	.956
Yes	1219 (44.4)	1169 (44.4)	50 (44.6)	
**Exp. of the lockdown, n (%)**				
Positive	596 (21.6)	569 (21.5)	27 (23.9)	
Neutral	1854 (67.3)	1791 (67.8)	63 (55.8)	
Negative	305 (11.1)	282 (10.7)	23 (20.3)	**.003**

For peer review only

Supplementary Table 3. Results of multivariable multinomial logistic regression investigating participants' characteristics associated with a positive ("not at all difficult") and negative ("very or extremely difficult") experience of the lockdown, using participants with a neutral ("slightly difficult") experience as reference group

	**Experience of the lockdown**					
	Positive (ref. neutral)			Negative (ref. neutral)		
	RRR	95% CI	P-value*	RRR	95% CI	P-value*
Socio-demographic						
**Gender**						
Men	ref.			ref.		
Women	**0.75**	**0.59 – 0.95**	**.016**	0.97	0.71 – 1.34	.873
**Financial difficulties**						
No	ref.			ref.		
Yes	1.08	0.84 – 1.40	.555	1.16	0.84 – 1.60	.353
Living environment						
**Living alone**						
No	ref.			ref.		
Yes	**1.93**	**1.52 – 2.46**	**<.001**	1.03	0.76 – 1.41	.836
**Home type**						
Apartment	ref.			ref.		
House	**1.49**	**1.03 – 2.16**	**.033**	0.67	0.34 – 1.32	.247
**Home characteristics**						
No balcony, terrace or garden	0.87	0.52 – 1.44	.578	0.63	0.32 – 1.25	.186
A balcony	ref.			ref.		
A terrace and/or a garden	1.05	0.79 – 1.40	.738	**0.66**	**0.44 – 0.99**	**.044**
Health status						
**Self-rated health**						
Very good / good	ref.			ref.		
Average / poor/ very poor	0.96	0.72 – 1.28	.761	1.23	0.88 – 1.74	.231
**Comorbidities**						
None	ref.			ref.		
1	0.79	0.62 – 1.01	.061	0.99	0.71 – 1.37	.952
2+	0.78	0.55 – 1.09	.144	0.71	0.46 – 1.08	.107
**BADL impairment**						
Independent	ref.			ref.		
Diff. / help for at least 1	1.13	0.80 – 1.58	.493	0.73	0.50 – 1.07	.104
**IADL impairment**						
Independent	ref.			ref.		
Diff. / help for at least 1	**0.78**	**0.61 – 0.99**	**.045**	1.40	1.00 – 1.98	.053
**Fear of falling**						
No	ref.			ref.		
Yes	**0.68**	**0.54 – 0.86**	**.001**	**1.52**	**1.07 – 2.15**	**.019**
**Memory difficulties**						
No	ref.			ref.		
Yes	1.04	0.74 – 1.47	.816	1.16	0.80 – 1.67	.440

For peer review only - http://bmjopen.bmj.com/site/about/guidelines.xhtml

**Depressive symptoms**							
No	ref.			ref.			
Yes	0.94	0.67 – 1.32	.737	1.44	0.99 – 2.10	.059
**Anxiety symptoms**							
No	ref.			ref.			
Yes	0.81	0.61 – 1.08	.151	1.13	0.79 – 1.61	.511
Social status							
**Feeling of loneliness**							
No	ref.			ref.			
Yes	**0.67**	**0.49 – 0.91**	**.011**	1.20	0.86 – 1.69	.281
**Socially supported**							
No	ref.			ref.			
Yes	**0.71**	**0.54 – 0.93**	**.012**	0.78	0.56 – 1.10	.158
**Emotional support**							
0	ref.			ref.			
1-2	1.05	0.65 – 1.71	.842	0.96	0.58 – 1.61	.889
3	1.36	0.89 – 2.08	.158	0.78	0.49 – 1.24	.293
**Previous participation in group activities**							
No	ref.			ref.			
Yes	**0.74**	**0.59 – 0.92**	**.007**	0.82	0.61 – 1.10	.193
**Having a dog**							
No	ref.			ref.			
Yes	1.05	0.67 – 1.64	.848	**0.32**	**0.11 – 0.90**	**.031**
**Unfamiliarity with technology**							
No	ref.			ref.			
Yes	**0.69**	**0.52 – 0.91**	**.008**	1.01	0.74 – 1.38	.952

Choice of variables: significant variables (threshold p<.05) in the bivariable multinomial logistic regression

Notes: N=2,246 complete cases (outcome distributed into 470/1,540/236)

RRR = relative risk ratio; CI = confidence interval. Reference: “slightly difficult”

* P-values from adjusted multinomial logistic regression.

For peer review only - http://bmjopen.bmj.com/site/about/guidelines.xhtml

Supplementary Table 3bis. Results from the multivariable multinomial logistic regression after multivariable imputation for missing data (sensitivity analysis).

	Experience of the lockdown					
	Not at all difficult (ref. slightly difficult)			Very or extremely difficult (ref. slightly difficult)		
	RRR	95% CI	P-value*	RRR	95% CI	P-value*
Socio-demographic						
**Gender**						
Men	ref.			ref.		
Women	**0.70**	**0.56 – 0.87**	**.001**	1.03	0.76 – 1.38	.858
**Financial difficulties**						
No	ref.			ref.		
Yes	1.10	0.87 – 1.38	.437	1.15	0.87 – 1.54	.326
Living environment						
**Living alone**						
No	ref.			ref.		
Yes	**1.90**	**1.52 – 2.37**	**<.001**	1.10	0.83 – 1.46	.519
**Home type**						
Apartment	ref.			ref.		
House	1.30	0.92 – 1.83	.131	0.70	0.37 – 1.32	.276
**Home characteristics**						
No balcony, terrace or garden	0.89	0.56 – 1.39	.596	0.62	0.33 – 1.13	.119
A balcony	ref.			ref.		
A terrace and/or a garden	1.10	0.85 – 1.42	.473	**0.62**	**0.43 – 0.90**	**.012**
Health status						
**Self-rated health**						
Very good / good	ref.			ref.		
Average / poor / very poor	0.99	0.77 – 1.29	.966	1.23	0.90 – 1.69	.198
**Comorbidities**						
None	ref.			ref.		
1	0.83	0.66 – 1.03	.094	1.03	0.76 – 1.40	.838
2+	0.89	0.65 – 1.21	.442	0.83	0.56 – 1.21	.325
**BADL impairment**						
Independent	ref.			ref.		
Diff. / help for at least 1	1.06	0.78 – 1.44	.713	0.77	0.55 – 1.10	.150
**IADL impairment**						
Independent	ref.			ref.		
Diff. / help for at least 1	**0.75**	**0.60 – 0.95**	**.016**	1.21	0.89 – 1.66	.227
**Fear of falling**						
No	ref.			ref.		
Yes	**0.73**	**0.59 – 0.90**	**.004**	**1.43**	**1.04 – 1.96**	**.029**
**Memory difficulties**						
No	ref.			ref.		
Yes	1.15	0.85 – 1.56	.366	1.09	0.77 – 1.53	.630
**Depressive symptoms**	ref.					
No	1.01	0.75 – 1.37		ref.	**1.16 – 2.31**	

For peer review only - http://bmjopen.bmj.com/site/about/guidelines.xhtml

	Yes			.950	**1.63**		**.005**
**Anxiety symptoms**							
	No	ref.			ref.		
	Yes	0.78	0.60 – 1.01	.062	1.04	0.75 – 1.44	.829
Social status							
**Feeling of loneliness**							
	No	ref.			ref.		
	Yes	**0.62**	**0.47 – 0.83**	**.001**	1.07	0.78 – 1.46	.678
**Usual social support**							
	No	ref.			ref.		
	Yes	**0.73**	**0.57 – 0.94**	**.014**	0.80	0.58 – 1.12	.191
**Emotional support**							
	0	ref.			ref.		
	1-2	0.91	0.59 – 1.39	.651	0.94	0.59 – 1.50	.798
	3	1.09	0.76 – 1.57	.646	0.77	0.51 – 1.18	.230
**Previous participation in group activities**							
	No	ref.			ref.		
	Yes	**0.79**	**0.65 – 0.97**	**.024**	0.83	0.64 – 1.09	.192
**Having a dog**							
	No	ref.			ref.		
	Yes	1.04	0.69 – 1.58	.849	**0.29**	**0.10 – 0.79**	**.016**
**Unfamiliarity with communication technology**							
	No	ref.			ref.		
	Yes	**0.66**	**0.52 – 0.85**	**.001**	1.12	0.85 – 1.49	.422

RRR relative risk ratio; CI confidence interval.

Reference: “slightly difficult” lockdown’s experience

* P-values from adjusted multinomial logistic regression.

For peer review only

For peer review only - http://bmjopen.bmj.com/site/about/guidelines.xhtml

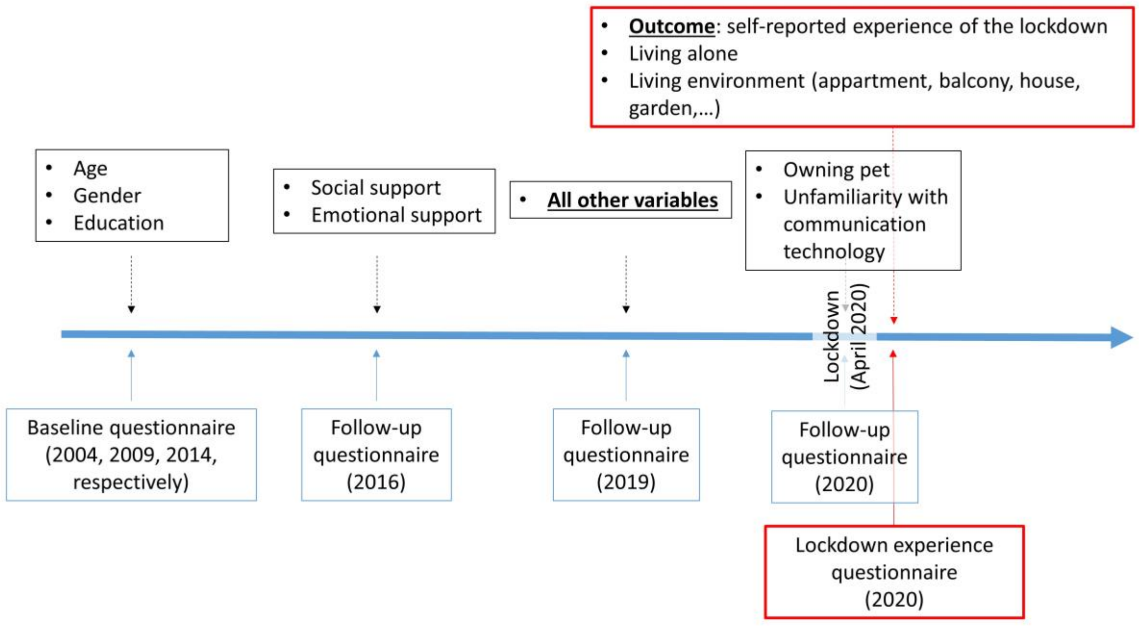

Supplementary Figure 1. Description of the sources of the study's variables and outcome

For peer review only - http://bmjopen.bmj.com/site/about/guidelines.xhtml
